# Impact of climate change on the potential global prevalence of *Macrophomina phaseolina* (Tassi) Goid. under several climatological scenarios

**DOI:** 10.3389/fpls.2025.1512294

**Published:** 2025-04-16

**Authors:** Peter F. Farag, Dalal Hussien M. Alkhalifah, Shimaa K. Ali, Aya I. Tagyan, Wael N. Hozzein

**Affiliations:** ^1^ Department of Microbiology, Faculty of Science, Ain Shams University, Abbasia, Egypt; ^2^ Department of Biology, College of Science, Princess Nourah bint Abdulrahman University, Riyadh, Saudi Arabia; ^3^ Department of Agricultural Microbiology, Faculty of Agriculture, Beni-Suef University, Beni-Suef, Egypt; ^4^ Department of Botany and Microbiology, Faculty of Science, Beni-Suef University, Beni-Suef, Egypt

**Keywords:** biogeography, DIVA-GIS, global warming, maxent, species distribution modeling

## Abstract

**Introduction:**

Climate change forms one of the most dangerous problems that disturb the earth today. It not only devastates the environment but also affects the biodiversity of living organisms, including fungi. *Macrophomina phaseolina* (Tassi) Goid. is one of the most pervasive and destructive soil-borne fungus that threatens food security, so predicting its current and future distribution will aid in following its emergence in new regions and taking precautionary measures to control it.

**Methods:**

Throughout this work, there are about 324 records of *M. phaseolina* were used to model its global prevalence using 19 environmental covariates under several climate change scenarios for analysis. Maximum Entropy (MaxEnt) model was used to predict the spatial distribution of this fungus throughout the world while algorithms of DIVA-GIS were chosen to confirm the predicted model.

**Results:**

Based on the Jackknife test, minimum temperature of coldest month (bio_6) represented the most effective bioclimatological parameter to fungus distribution with a 52.5% contribution. Two representative concentration pathways (RCPs) 2.6 and 8.5 of global climate model (GCM) code MG, were used to forecast the global spreading of the fungus in 2050 and 2070. The area under curve (AUC) and true skill statistics (TSS) were assigned to evaluate the resulted models with values equal to 0.902 ± 0.009 and 0.8, respectively. These values indicated a satisfactory significant correlation between the models and the ecology of the fungus. Two-dimensional niche analysis illustrated that the fungus could adapt to a wide range of temperatures (9 °C to 28 °C), and its annual rainfall ranges from 0 mm to 2000 mm. In the future, Africa will become the low habitat suitability for the fungus while Europe will become a good place for its distribution.

**Discussion:**

The MaxEnt model is potentially useful for predicting the future distribution of *M. phaseolina* under changing climate, but the results need further intensive evaluation including more ecological parameters other than bioclimatological data.

## 1 Introduction

*Macrophomina phaseolina* (Tassi) Goid. is one of the most devastating necrotrophic, seed- and soil-borne pathogenic fungus that belongs to the Botryosphaeriaceae family (Dell’Olmo et al., 2022; Ortiz et al., 2023). It infects more than 800 plant species over the world, including economically important crops such as *Glycine max* (L.) Merr. (soybean), *Helianthus giganteus* E. Watson (sunflower), *Vicia faba* L. (bean), *Gossypium hirsutum* L. (cotton), *Sesamum indicum* L. (sesame), and cereal plants causing charcoal rot, dry root rot, wilt, blight, and damping-off diseases (Farr and Rossman, (2022); Tančić Živanov et al., 2019; Kaur et al., 2023). This fungus is characterized by forming a spherical aggregating mass of hyphae called microsclerotia, which can survive up to 15 years in soil and crop debris as a resistant structure to overcome several inadequate environmental conditions, making disease control a challenge (Marquez et al., 2021).

Climate has a major role in the prevalence of *M. phaseolina* which is thermophilic in nature and reflects a critical correlation with soil and environmental factors (Bashir, 2017). For disease occurrence, high temperature and low moisture played a pivotal role in the development and distribution of this fungus, where maximum disease was observed at 25-32°C air and 23-35°C soil temperature (Bashir, 2017; Marquez et al., 2021). Some other factors are also responsible for the occurrence of disease such as different pathogen strains, inconsistency in disease resistance and susceptibility, and soil physical and chemical characteristics that alter the interaction between pathogen and host (Bashir, 2017).

Generally, *M. phaseolina* is geographically distributed in tropical and sub-tropical areas with semi-arid weather (Wrather et al., 2001; Sarr et al., 2014). Despite the fact that *M. phaseolina* is a disease that thrives in warm climates, it has been observed in recent years to spread in a number of different locations and is now ubiquitous all over the world (Veverka et al., 2008). According to the Intergovernmental Panel on Climate Change (IPCC), one of the primary reasons for its appearance is climate change. According to the IPCC, global warming is likely to continue, and the average temperature of the earth’s surface is expected to rise by 0.3–4.5 degrees Celsius (Chen et al., 2022). Thus, this phenomenon has disparate effects on biodiversity and has altered fungal attributes, resulting in yield losses and economic damage (Ghini et al., 2012; Dell’Olmo et al., 2022). Nowadays, the fungus can adapt to various agroecological conditions, and its aggressivity fluctuates depending on different environmental factors (biotic and abiotic) and geographic areas (Mihail and Taylor, 1995; Fang et al., 2011; Borer et al., 2016).

Climate change has introduced additional hurdles in safeguarding crops from fungal infections, jeopardizing food security. Understanding fungal dispersal and predicting appropriate future environments are essential for implementing preventative measures for its prevention and control (Dietzel et al., 2019; Savary et al., 2019). Examining the spatial distribution of infections yields critical insights into their prevalence and the influence of environmental conditions on phytopathogens and epidemics. A variety of spatial statistical approaches have been employed to characterize the spread of fungal diseases and affected plants (Wu et al., 2001; Taliei et al., 2013). The clarification of the present distribution status of *M. phaseolina* in relation to climate change and the constraints of current mitigation efforts is crucial since there are numerous opportunities for future research on this fungus (Pandey and Basandrai, 2020).

Geographic Information Systems (GIS) facilitate the mapping of pathogen distribution and the spatial modeling of environmental factors influencing disease occurrence by describing, analyzing, and visualizing data associated with geographic coordinates (Fischer and Nijkamp, 1992; Murad and Khashoggi, 2020). It is regarded as an advantageous instrument for forecasting species proliferation and assessing infestation impacts (Alkhalifah et al., 2022). Conserving species in their native habitats needed comprehension of their spatial distribution patterns and ecological interconnectedness. Distribution Models (SDMs) integrate geographical data from various sources utilizing GIS tools (Balakrishnan et al., 2018).

Species distribution modeling (SDM) is a crucial technique that delineates the specific habitat of each species (Runquist et al., 2019; Deneu et al., 2022). In recent years, numerous modeling software applications utilizing various mathematical methods have been created to achieve this objective; however, MaxEnt (Maximum Entropy Model) and DIVA-GIS are the most effective and precise tools employed to assess the impact of climate change on diverse fungal species. CLIMEX, GARP, and HABITAT are widely utilized methods for assessing the future distribution of certain species in response to climate change; nonetheless, their efficacy has been noted to be inferior to that of MaxEnt (Shabani et al., 2014; Hosni et al., 2020; Zurell et al., 2020).

This study aims to examine the effects of climate change on the worldwide occurrence of *Macrophomina phaseolina* (Tassi) Goid., a critical soil-borne fungal disease that jeopardizes food security in many crops. This research aims to estimate the current and future distribution of *M. phaseolina* under various climatological scenarios by leveraging a comprehensive dataset of occurrence records of this pathogen and employing species distribution modeling techniques, notably the Maximum Entropy (MaxEnt) model. The research aims to discover critical environmental factors affecting the habitat appropriateness of the fungus and to evaluate how shifting climate conditions may impact its regional distribution. This study seeks to address the crucial question: How will climate change influence the distribution and prevalence of *Macrophomina phaseolina* in the forthcoming decades?

## 2 Materials and methods

### 2.1 Global occurrence data collection for *M. phaseolina*

**Figure 1:**
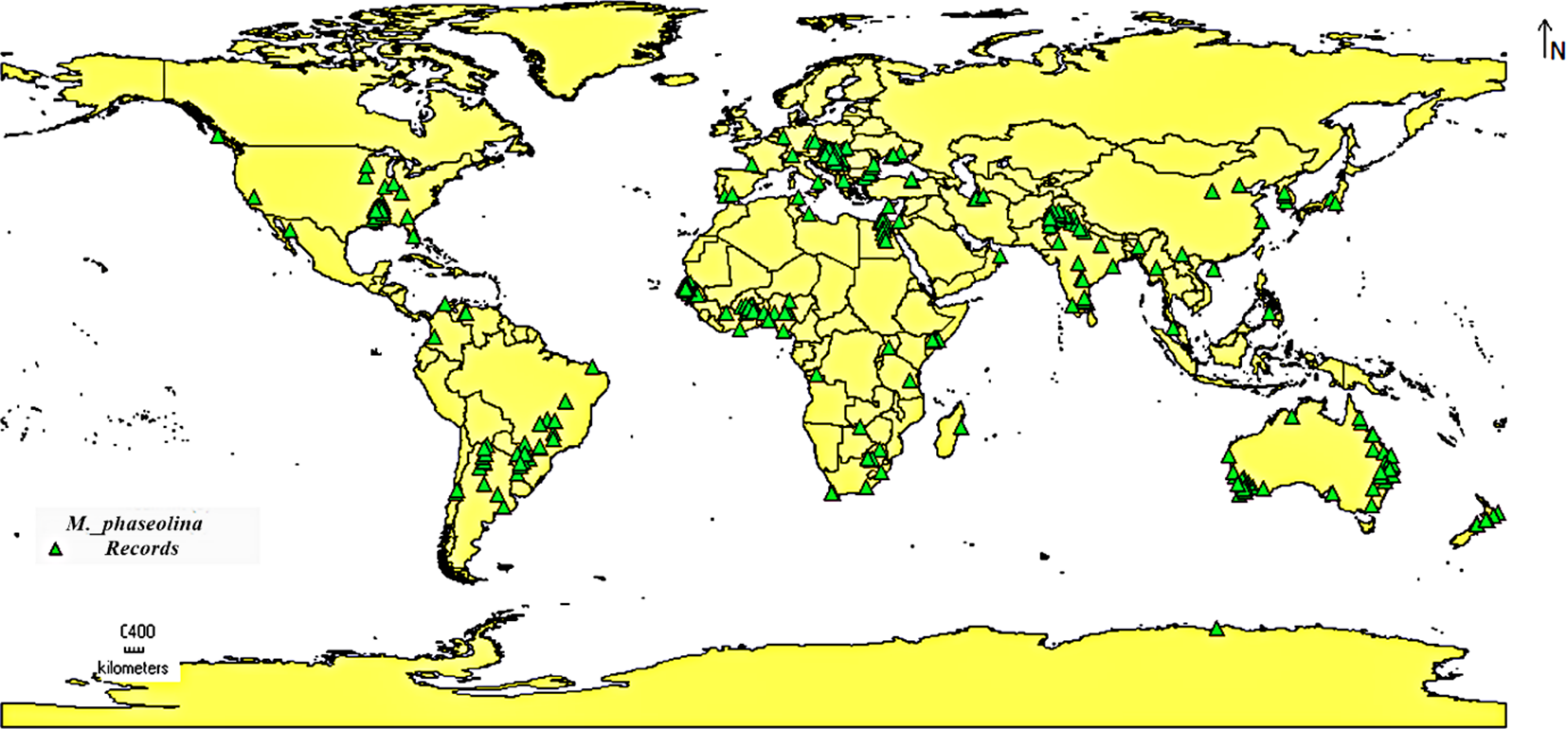
Global prevalence of *M. phaseolina* according to the collected occurrence data.

In this study, most of the current distribution records of *M. phaseolina* were gathered from published scientific literature, and the rest of the occurrence data were obtained from the Global Biodiversity Information Facility (GBIF) digital databases (GBIF.org, 2022). After choosing the precise location data and removing the duplicated and high spatial uncertain records (data points characterized by a considerable degree of uncertainty concerning their geographic coordinates. This uncertainty may stem from multiple reasons, including: Inaccurate Location Data: The supplied coordinates may not accurately represent the true location of the event; and Low Precision: The information may rely on general geographic descriptors instead of precise coordinates, resulting in uncertainty), a total of 324 geo-referenced coordinates (**Supplementary Table S1**) were saved as comma delimited (CSV) Excel format and used for species distribution modeling (SDM) analysis (**Figure 1**).

### 2.2 Bioclimatological covariates

Nineteen bioclimate covariates were downloaded from the global WorldClim database (www.worldclim.org, accessed on 6 Feb 2023) to start the species distribution modeling at a spatial resolution of around 5 Km^2^ (**Supplementary Table S2**). These variables were generated using the average interpolated climate data from 1950 to 2000 (Hosni et al., 2022). Layers of bioclimatic variables 8-9 and 18-19 were dislodged to establish the current climatic data due to their spatial distortions in those variable layers (Samy et al., 2016; Hosni et al., 2020). So, only 15 bioclimatic covariates were transformed into ASCII files using ArcGIS version 10.7. Pearson correlation coefficient has been applied to exclude extremely correlated covariates (r2 ≥ |0.8|) and minimize multicollinearity from distribution models (Wang et al., 2018; Hosni et al., 2022). According to this, only five climatic variables were selected to establish final models: Bio_6, Bio_1, Bio_4, Bio_12, and Bio_10.

We modeled the future distribution patterns for *M. phaseolina* to investigate the variability of their potential habitat across several climate scenarios (Saha et al., 2021). The potential values for climatic covariates under future climate conditions in the 2050s (average estimation between 2041 and 2060) and 2070s (average estimation between 2061 and 2080) were derived from the global climate model (GCM) [MRI-CGCM3, Code MG] developed by the Meteorological Research Institute under two IPCC-CMIP5 [Coupled Model Intercomparing Project Phase 5] representative concentration pathways (RCPs), 2.6 and 8.5 (https://www.worldclim.org/data/cmip6/cmip6climate.html (accessed on 5 Feb 2023). RCP 2.6 is the minimum greenhouse gas emission scenario, while RCP 8.5 is the maximum greenhouse gas emission scenario (Heringer et al., 2019; Mohammadi et al., 2019).

### 2.3 Distribution modeling procedures

Two modeling software: the maximum entropy (MaxEnt, version 3.4.1) and Clim. Model on DIVA-GIS software V7.5 were utilized to predict the suitable habitat of *M. phaseolina* (Phillips et al., 2022; Phillips and Dudík, 2008). Both used presence-only and small sample-size data to forecast species distribution and model habitat suitability as a function of environmental variables with pseudoabsence points (Since the models are using presence-only data, pseudoabsence points are artificially created locations where the species is assumed to be absent. These points help balance the dataset and provide a reference against which to compare the present data, allowing the model to better understand the conditions under which the species thrives) (Bradie. and Leung, 2017). To evaluate the predictive performance of our models, species occurrence information for model calibration was divided into a training set (75% of occurrence records) and a test set (25% of occurrence records), where this process was repeated five times as a choose in Maxent option (Hosni et al., 2022). We followed (Tavanpour et al., 2019). for settings and used 10000 maximum random background points as pseudoabsence, regularization multiplier 1, 10000 maximum iterations with 10^-5^ convergence threshold, and selecting the logistic output format. The habitat suitability areas of the resultant models were classified into 5 classes (not suitable, low, medium, high, and very high). Also, the Diva-GIS modeling tool was used to generate the limitation factor map. This map is a crucial component that helps identify and understand the factors that limit the distribution of the species. It provides valuable information about the environmental variables or conditions that influence the presence or absence of a species in a given area.

### 2.4 Model interpretation and evaluation

The developed model was evaluated by calculating the area under the curve (AUC) of the receiver operating characteristic (ROC) plot, which varied from 0 (which corresponded to a random distribution) to 1 (which represented a perfect prediction) (Phillips et al., 2006; Mulieri and Patitucci, 2019). Models that have analysis of variance (AUC) values that are greater than 0.9 suggest outstanding prediction accuracy, while values that fall between 0.7 and 0.9 indicate good prediction accuracy, and values that are less than 0.7 indicate low prediction accuracy (Pearson, 2010). True Skill Statistics (TSS) was utilized in order to evaluate the predictability of the models that were projected, and the values that were utilized varied from -1 to 1 (Allouche et al., 2006). Negative numbers that are near to 0 suggest a weak association between the prediction model and the distribution, whereas positive values that are close to 1 show a significant relationship between the two. Furthermore, the jackknife test was utilized in structural equation modeling (SDM) to examine the impact of dominant environmental variables on model outcomes in order to choose dominant elements (Allouche et al., 2006; Yang et al., 2013).

## 3 Results

### 3.1 Model performance

**Figure 2:**
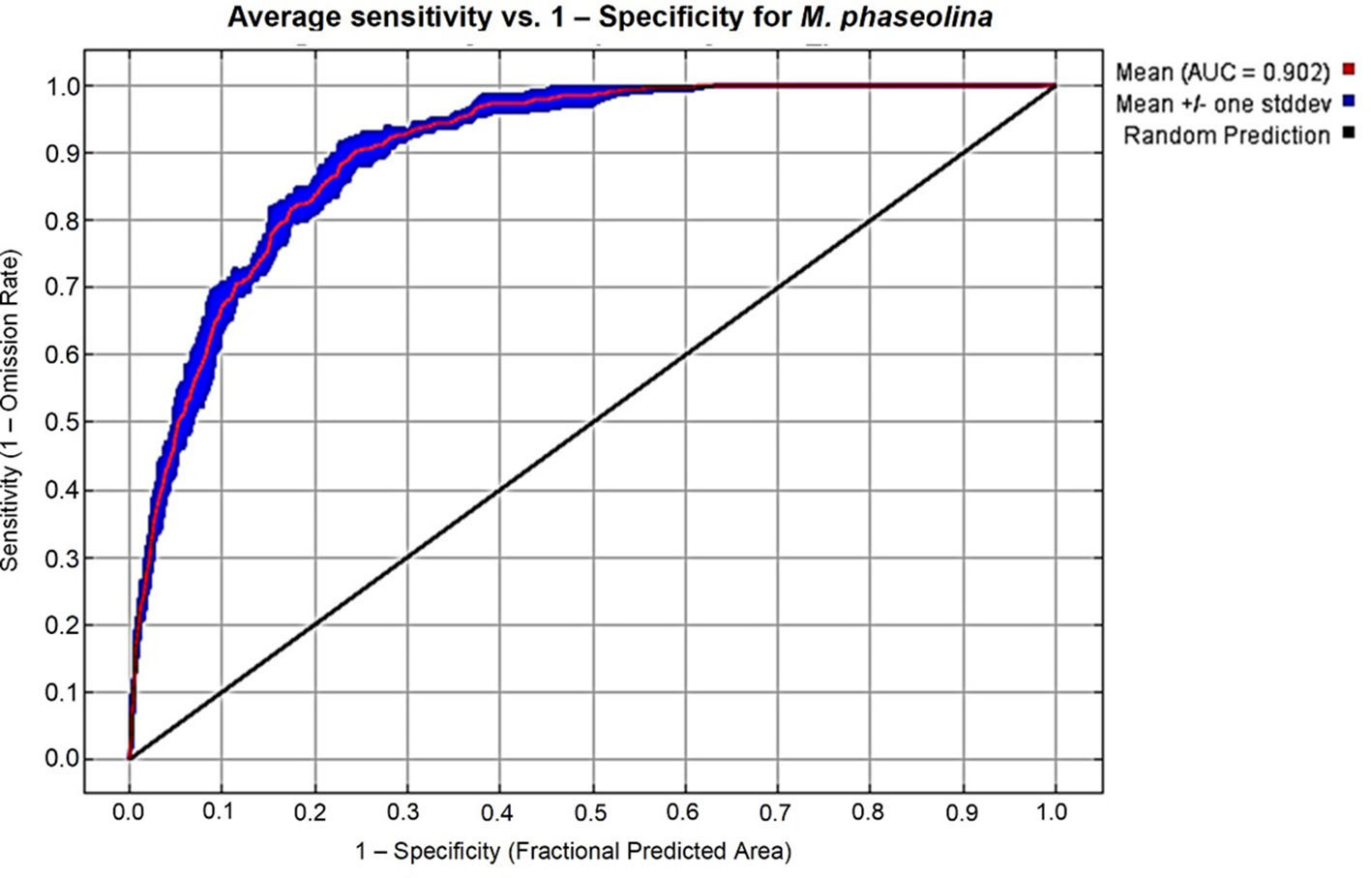
ROC curve and AUC value for the current period over the replicate runs.

AUC values were used to evaluate the performance of the MaxEnt model. In our study, calibration of the model for *M. phaseolina* was satisfactory (AUC_mean_ = 0.902 ± 0.009, **Figure 2**). This finding means that *M. phaseolina* current distribution characterized by the selected variables is excellent. The functional assessment of this model was supported by TSS where its value equals 0.8. This value represents a good quality modeling process, knowing that the acceptable TSS value is ≥ 0.5.

### 3.2 Contribution and effects of bioclimatic covariates

**Figure 3:**
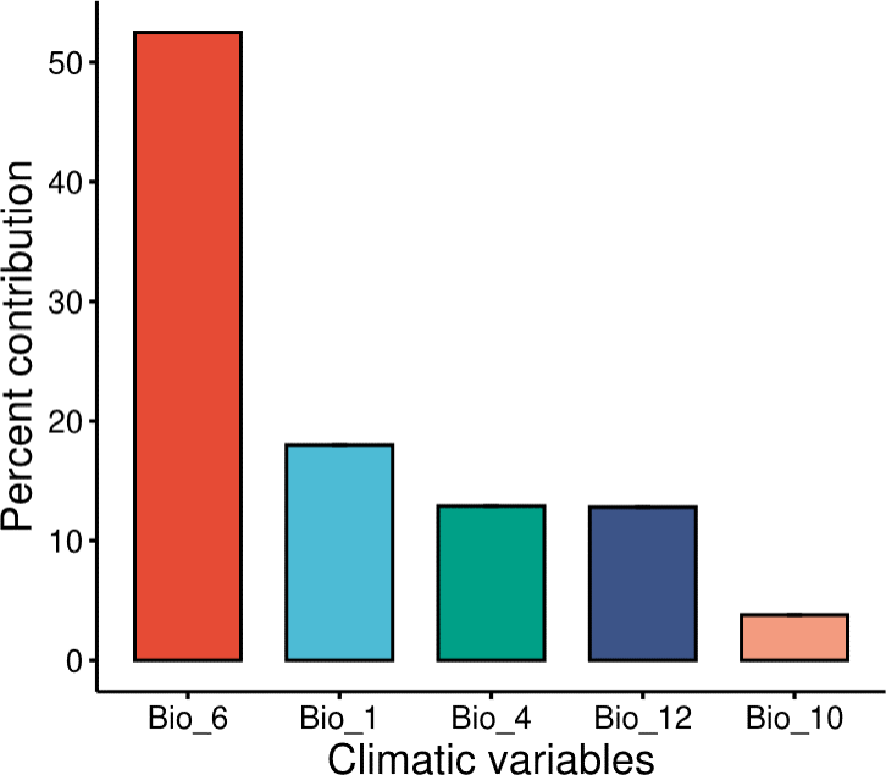
Estimates of contribution percentage to species distribution for the most relevant climatological variables. Bio_6 is the minimum temperature of coldest period; Bio_1 is Annual mean air temperature; Bio_4 is the Temperature seasonality; Bio_12 is Annual precipitation; Bio_10 is the Mean temperature of the warmest quarter.

**Figure 4:**
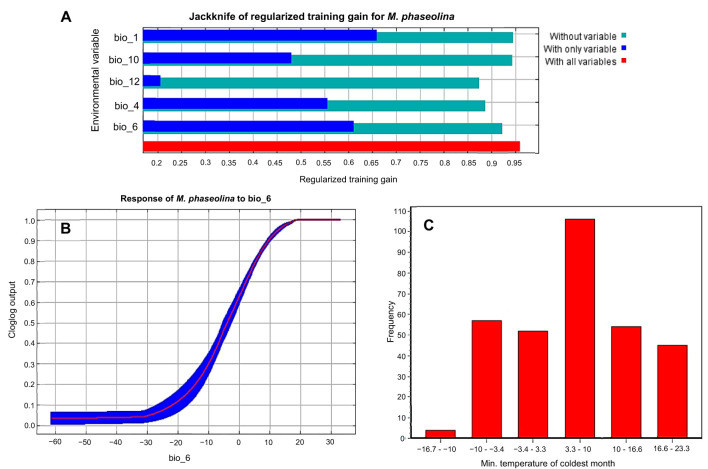
Selected climatic covariates: **(A)** The jackknife test of the five most important variables, **(B)** Response curve of the most effective climatic parameter (bio_6) on fungus distribution, **(C)** Frequency analysis of the distribution range of records against Bio_6.

Following the removal of the other factors that were linked with *M. phaseolina*, the jackknife test was used to determine the percentages of contribution that each of the five most important climatological variables (bio_1, bio_10, bio_12, bio_4, and bio_6) had for the predictions of *M. phaseolina* distribution (**Figure 3**, **4B**). According to the results of this test, the climatic parameter that had the greatest impact on the distribution of fungi was the Minimum Temperature of the Coldest Month (bio_6), which had a value of 52.5%, followed by the annual mean temperature (Bio_1) with 18% contribution. The other bioclimatic factors, according to the jackknife test, were bio_4, bio_12, and bio_10 showed the lowest contribution percentage, respectively (**Figures 3**, **4A**). According to the response curves and the frequency of the bioclimatic variables that contributed the most to the fungus’s favorable bio_6 (**Supplementary Figures S1**, **S2**), the temperature range that the fungus thrived in was between 3.3 and 10 degrees Celsius (**Figures 4A–C**).

### 3.3 Limitation factor map

**Figure 5:**
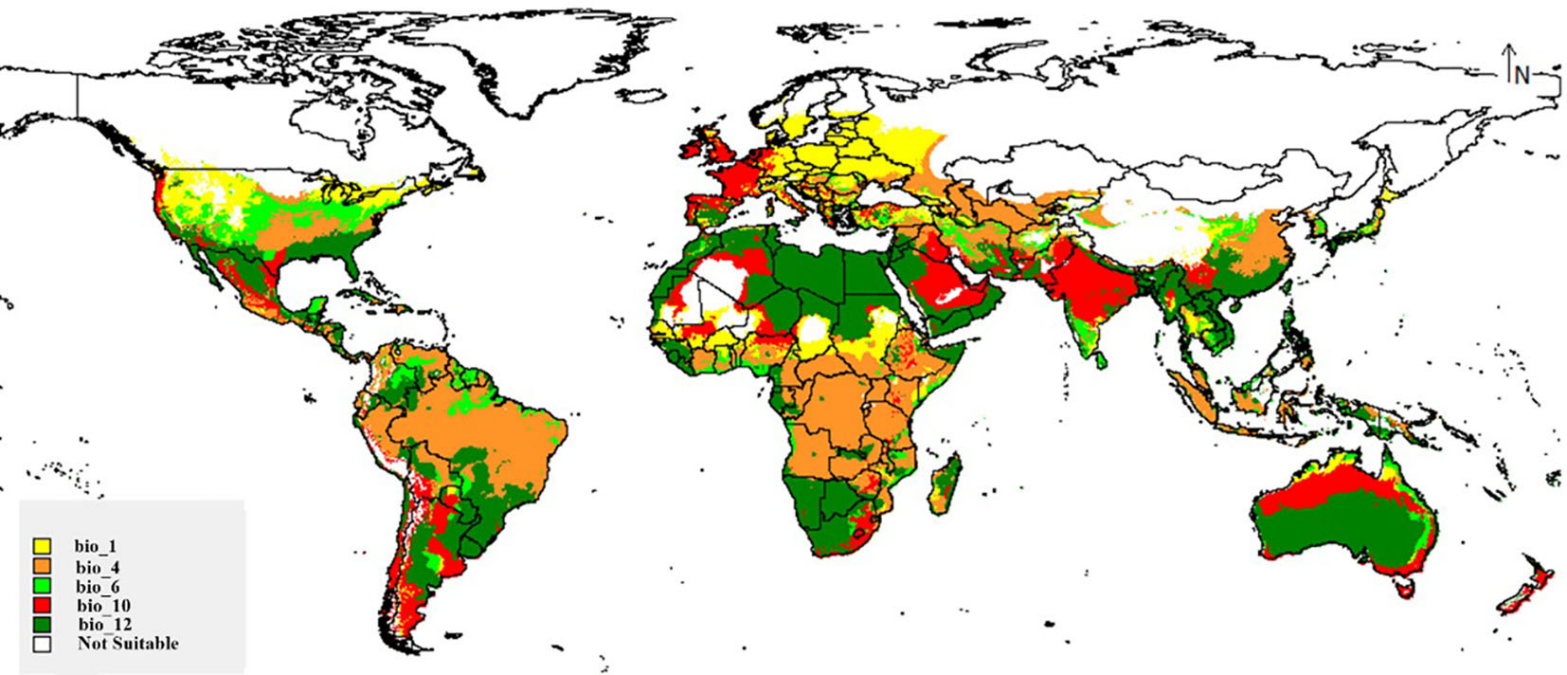
Map showing key limiting factors for global distribution of *M. phaseolina*.

**Figure 5** depicts the limiting effects of the fungus through its range. On the map, in the Middle East and Australia, the fungus distribution is affected by the bio_12 (Annual precipitation) as a limitation factor, especially drought through these deserts. Temperature seasonality (bio_4) limits the existence of *M. phaseolina* in Brazil, the tropical zone of Africa, and parts of Southeast Asia; very high temperatures there could form a limitation to this species. In Europe, the fungus is largely influenced by the low values of the average annual temperature (bio_1). These three factors form the main limitation factor through the wide range of *M. phaseolina*.

### 3.4 Two-dimensional niche analysis

**Figure 6:**
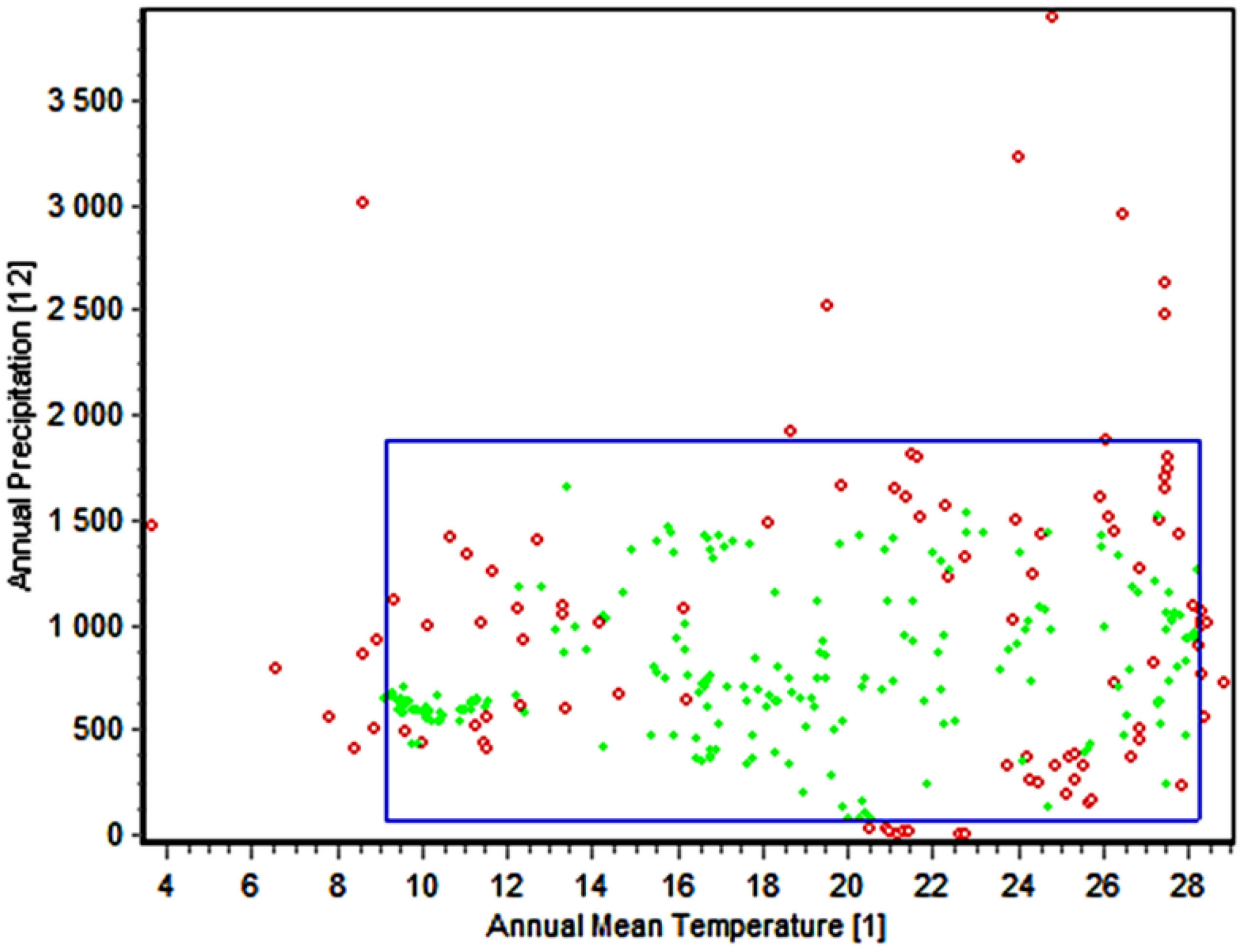
The two-dimensional niche of *M. phaseolina* between environmental covariates bio_1 (red dots) and bio_12 (green dots). The blue box represent envelope of these to variables (the range that this species could live within)The green dotes indicated the homogeneity of this points and occurrent of it on the enveloped of this species even for all 19 bioclimatic variables while the red dotes indicate the occurrence of this points outside the enveloped either for the tested variables (Bio_1 & Bio 12) or one or more other bioclimatic variables.

The present study utilized the enveloped test to create the 2D niche of *M. phaseolina* based on the most significant environmental variables used in examining this fungus. The test was conducted between the annual mean temperature (bio_1) and annual precipitation (bio_12) (**Figure 6**). The results demonstrated a broad spectrum of adaptability to varying environmental conditions, with 318 observations, of which 287 (90.3%) fell within this range. The yearly temperature fluctuates between 9°C and 28°C, while the annual precipitation varies from 0 mm to 2000 mm. The observed outcome from the frequency influence of the minimum temperature during the coldest month on the fungus confirms its remarkable adaptability to a broad spectrum of cold temperatures, ranging from 3.3°C to 10°C (**Figure 4C**). These observations shed light on the broad prevalence of this fungus as it can grow in dry, hot deserts and cold, rainy regions.

### 3.5 Current prediction of the potential distribution of *M. phaseolina*

**Figure 7:**
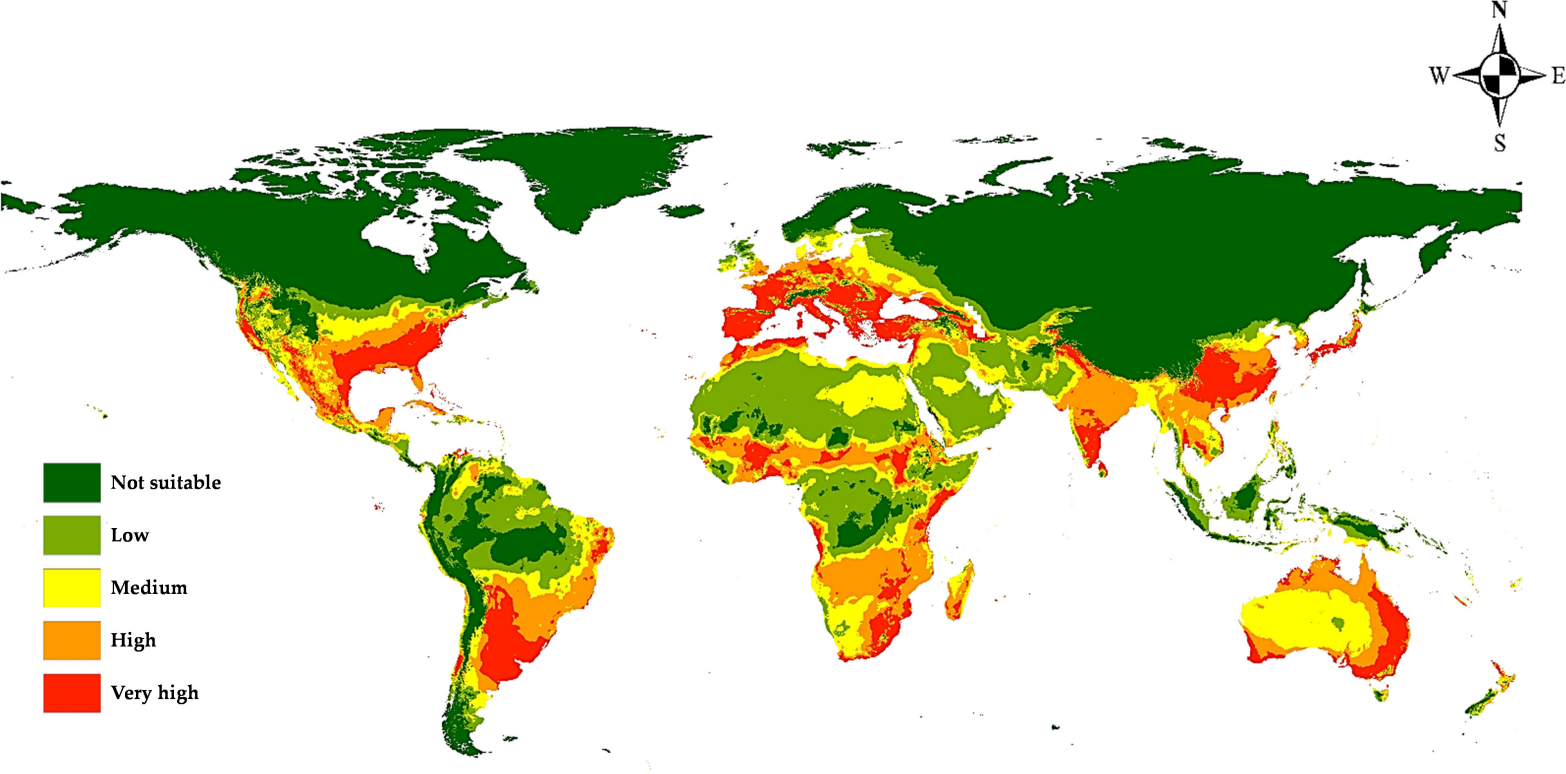
Predicted current potential global distribution of *M. phaseolina* using MaxEnt.

**Figure 8:**
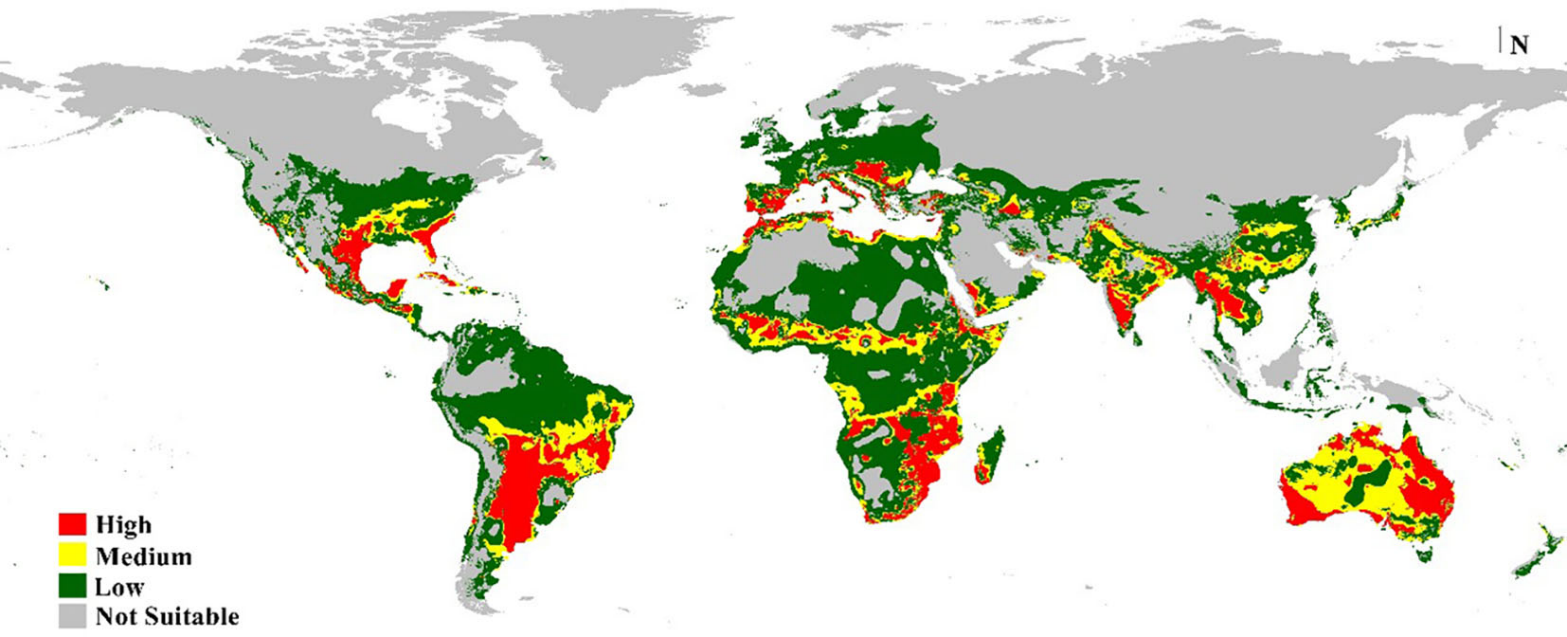
Predicted current potential global distribution of *M. phaseolina* using Bioclim DIVA-GIS.

According to the distribution points and environmental variables, the current models generated by MaxEnt and DIVA-GIS showed compatible habitat suitability and agreed with the actual collected distribution data of *M. phaseolina* (**Figures 7**, **8**). This fungus is an ecumenical cosmopolitan species that inhabits all continents. On the basis of the map, we are able to draw the conclusion that the only regions that appear to be exempt from the invasion of this fungus are those that have extremely cold weather or very dry, hot desert places. This is the case in the Sahara Desert of Africa and the Middle East, as well as in colder countries such as the northern sections of Russia and Canada. There is a wide variety of habitats that are suitable for the different regions of the planet. In Europe, the models showed that the habitat appropriateness for the fungus was extremely high and good across the entire territory, which included Spain, Italy, Turkey, Greece, and Germany. On the other hand, the northern east territories of Europe exhibited habitat suitability that was low to medium. The eastern coast and small parts of the western coast of Australia also showed high and very high risk, while its central part showed medium climate suitability. Meanwhile, New Zealand followed the same pattern. Africa had low-to-moderate climate appropriateness across the majority of the continent, with elevated and extreme hazards in the central to northern regions and southern nations of the continent. Also, small areas of Horn Africa, such as Somalia appeared highly suitable. Moreover, in Asia, the risk is high to very high mainly through China and India. In North America, the resulting current models indicated low suitability of *M. phaseolina* distribution over its land, except for the eastern coast of the United States and the western coast of the Mexican Gulf, which showed very high (excellent) suitability. Finally, South America appeared to have very high suitability in Brazil and Chile.

### 3.6 Future prediction of the potential distribution of *M. phaseolina*

**Figure 9:**
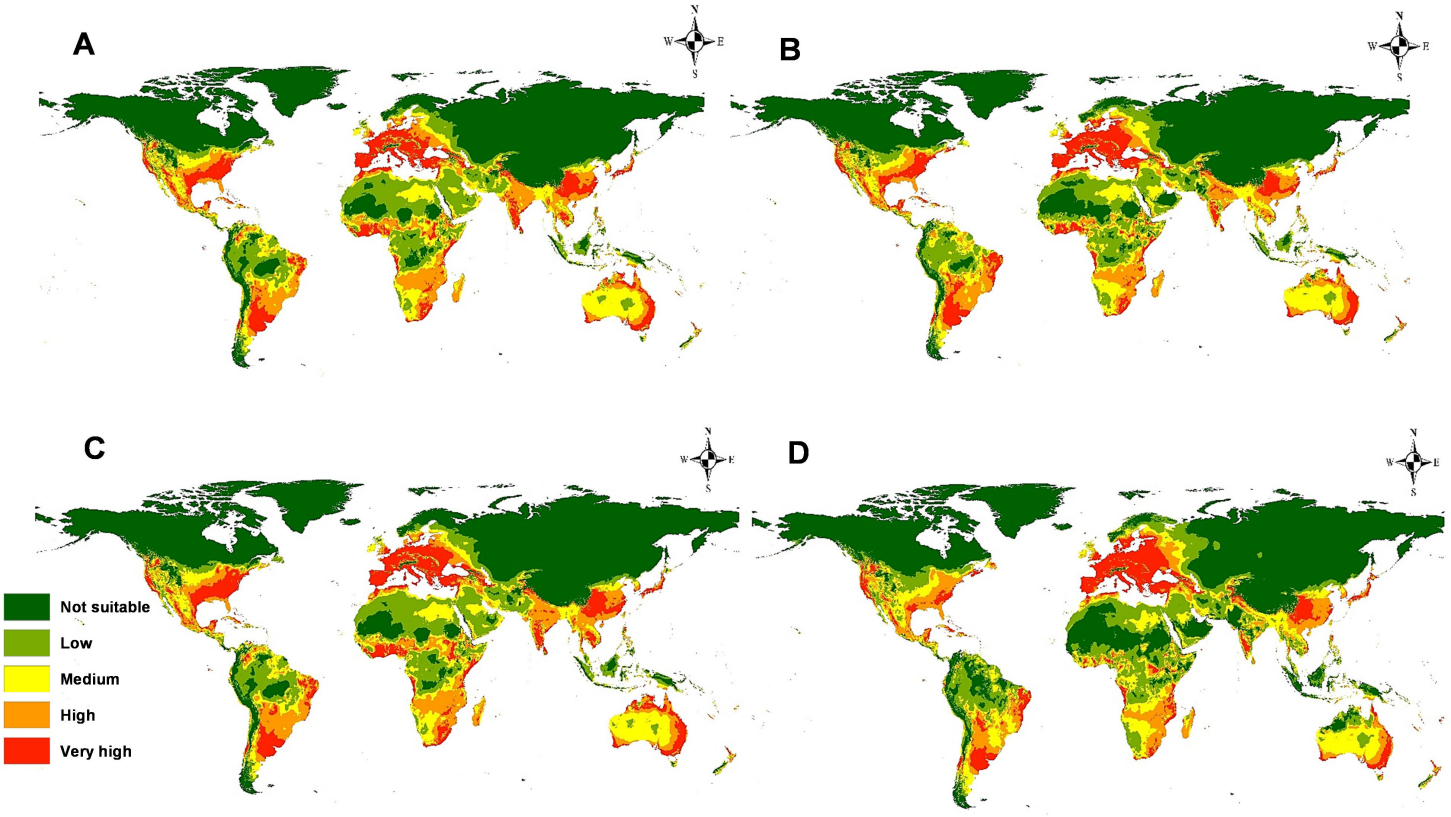
Models of predicted global future distribution of *M. phaseolina* under two representative concentration pathways (RCPs): **(A)** 2050 for RCP 2.6; **(B)** 2050 for RCP 8.5; **(C)** 2070 for RCP 2.6, and **(D)** 2070 for RCP 8.5.

The predictive models for the potential spread of *M. phaseolina* under four future climate change scenarios RCP 2.6 and RCP 8.5, for the years 2050 and 2070, are illustrated in **Figure 9**. For RCPs 2.6 and 8.5 during the period 2050 (**Figures 9A–C**), the most affected continents by the prevalence of the fungus are Europe and South America. The eastern region in Europe, including Russia, and northern areas in South America will become high and very high habitat suitability. Other continents showed no great differences in the pathogenicity of the fungus in the future. On the contrary, the virulence of this fungus will decrease and can’t invade some territories of Africa. For RCPs 2.6 and 8.5 during period 2070 (**Figures 9B–D**), the predictive models illustrated a dramatic change in the fungus distribution, where there is a noticeable decrease in the spreading of the fungus in most regions in Africa, India, and the United States compared to the current status and period 2050. On the other hand, the continued increase of its distribution in Eastern Europe.

**Figure 10:**
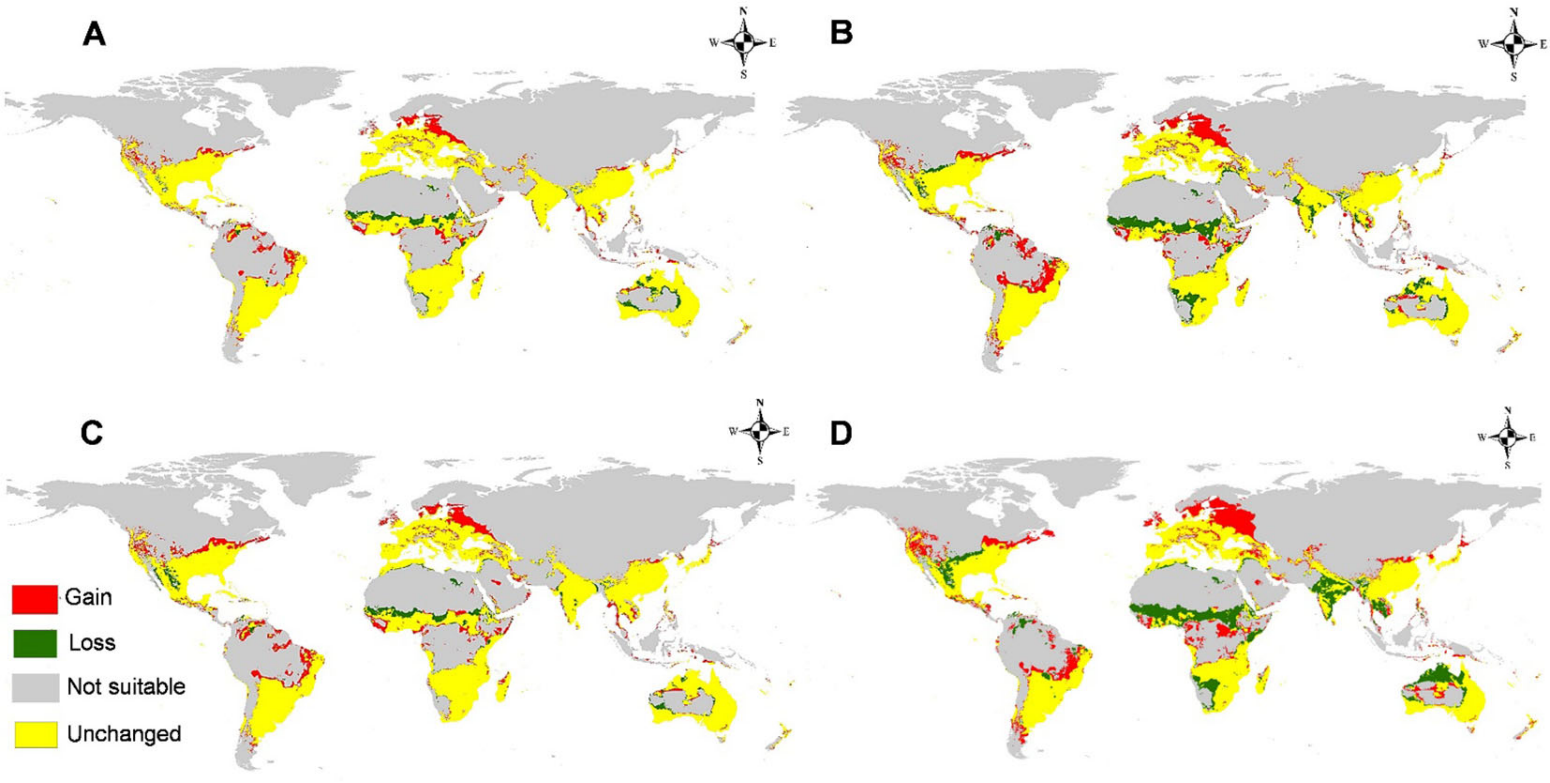
Calibration maps illustrate gain and loss in habitat suitability of *M. phaseolina* through the studying future scenarios against the current status with a threshold (>0.5): **(A)** 2050 for representative concentration pathway 2.6 (RCP 2.6); **(B)** 2050 for RCP 8.5; **(C)** 2070 for RCP 2.6, and **(D)** 2070 for RCP 8.5.

The calibration maps of current and future forecasts for two different RCPs in 2050 and 2070 are used to summarize the level of changes in *M. phaseolina* distribution owing to global warming (**Figure 10**). Under low presumptive emissions of greenhouse gases (GHG) (RCP 2.6 in 2050 and 2070), the changes are slightly notable and usually not significant on all continents. However, the fungus will lose some of its habitats, especially in areas of Africa, and lose its habitat in India and Australia for the period 2050 than 2070 (**Figures 10A–C**). Moreover, for the highest presumptive emissions of GHG (RCP 8.5 in 2050 and 2070), the fungus will lose its habitat suitability, especially in the equatorial and tropical regions in Africa, India in Asia, and north parts of Australia, while there is a clear gain in suitability appears in Eastern Europe (**Figures 10B–D**).

## 4 Discussion

Undoubtedly, climate change has become the center of world attention in recent years. This phenomenon affects the biodiversity of living organisms, including fungi, leading to the extinction of some species, and increasing the aggressivity of others (Sax et al., 2013; Nnadi and Carter, 2021; Mora et al., 2022). One Health (OH) is the concept that the health of humans, animals, plants, and their shared ecosystem is inextricably linked (Centers for Disease Control and Prevention (CDC), 2022). This approach tackles burgeoning problems such as food safety (Garcia et al., 2020). Plants supply over 80% of the food consumed by humans and are the main source of nutrition for livestock, however, plant diseases often threaten the availability of plants for humans and animals (Savary et al., 2017; Food and Agriculture Organization of the United Nations (FAO), 2020). In addition, agricultural crops may act as carriers of many human pathogens and harmful fungal-based toxins, making these plants the main origin of foodborne outbreaks (Rizzo et al., 2021). Increasing the studies in these fields will demonstrate the value of the OH approach for the perception and mitigation of the negative impacts of these issues.

*Macrophomina phaseolina* can cause substantial yield losses in crops such as *Glycine max* (L.) Merr. (soybean), *Sorghum bicolor* (L.) Moench (sorghum), and several cereals crops under high temperatures and low soil moisture (below 60%), impacting the incomes of farmers and threatening global food security (Kaur et al., 2012; Ghosh et al., 2018). Also, this fungus produces several types of mycotoxins, such as phaseolinone, mullein, kojic acid, and moniliformin, which negatively impact food safety for humans and animals (Khambhati et al., 2020). So, studies on species range changes in the near and long future are crucial for implementing effective management measures and conserving valuable species.

The present study forms a step for better elucidation of the habitat requirements of *M. phaseolina* and how it will respond to climate change. The results showed that the choice of environmental variables has a certain effect on the prediction of niche models. Many researchers who use the MaxEnt model to predict the distribution of species non-selectively use all the environmental factors or most environmental factors. The environmental variables, sourced from the WorldClim database, are derived from temperature and precipitation data tailored to the specific requirements of occurrence calculations. Consequently, there are unavoidable correlations between the autocorrelation of these variables and other matters (Merow and Silander, 2014; Remya et al., 2015; Yi et al., 2016). The predominant method employed for assessing model accuracy is the ROC curve method (AUC method), which is now acknowledged as a specialized model evaluator. It delivers performance evaluation data across all threshold ranges, as it is unaffected by diagnostic thresholds (Wang et al., 2018). The generated habitat suitability of our models coincided closely with the actual occurrence of fungus records with a high AUC value equal to 0.902, implying a close association between the model and the species’ ecology. Furthermore, the TSS value of 0.8 indicated that the model predictions and the dispersion of the fungus were in perfect accord (Naeem et al., 2018).

The most important influencing parameter to *M. phaseolina* distribution is the temperature, agreed with previous studies in microbial and biological organisms (Makori et al., 2017; Sohail et al., 2020). Temperature is a key factor in fungal growth dynamics and understanding the effect of temperature on fungal growth is an essential part of fungal physiology (Ancin-Murguzur et al., 2018; Mustafa et al., 2023). The majority of fungi are mesophilic where they can grow at temperatures within the range from 15°C to 37°C with an optimal temperature of 25–30°C (Aguilar-Paredes et al., 2023). Understanding the impact of temperature on fungal communities will aid the design of effective management strategies against climate change and associated microbial risks (Ibáñez et al., 2023).

Here are a few essential considerations emphasizing the significance of limitation factor maps in SDM (Warren et al., 2008; Elith et al., 2010): Limitation factor maps assist in identifying the principal environmental factors that affect species dispersal. Through the examination of the correlation between species occurrence data and environmental variables, researchers can identify the most significant elements influencing habitat suitability for species. Incorporating limiting constraints into species distribution models enhances their accuracy and predictive capability. III) They offer insights on the ecological necessities and tolerances of a species. By delineating the variables that constrain its spread, scientists can enhance their comprehension of the species’ niche and the spectrum of environmental conditions in which it can endure. This knowledge enhances our comprehensive understanding of species-environment interactions and aids in evaluating species’ responses to environmental alterations. IV) They serve as essential instruments for conservation planning and management. They can assist in identifying regions with high habitat appropriateness for a species, places presently unsuited but capable of becoming suitable with appropriate interventions, and regions expected to remain unsuitable in the future due to constraints imposed by specific causes. This information is essential for prioritizing conservation initiatives, identifying crucial habitats, and executing successful management techniques (Moritz et al., 2012; Nabil et al., 2020).

The latest prediction map (**Figure 7**) indicates the global dissemination of *M. phaseolina*, corroborating numerous prior studies on its ecology and distribution (Marquez et al., 2021). Western Europe exhibits high to extremely high habitat suitability, whilst the colder nations in Eastern Europe demonstrate low suitability. This may result from the snowy conditions that inhibit fungal growth. Numerous studies have examined the impact of temperature on fungal development (Cesondes et al., 2007; Das et al., 2008; Cesondes et al., 2012). In Africa, the fungus exhibits exceptional climate suitability in equatorial, subequatorial, and tropical regions, where it endures elevated temperatures due to microsclerotia (Manici et al., 1995; Akhtar et al., 2011; Lodha et al., 2022). Sahara deserts and regions with elevated precipitation levels have limited habitat appropriateness, with insufficient soil moisture identified as a significant predisposing factor for the fungus (Goudarzi et al., 2008; Lodha and Mawar, 2020). India, China, and Southeast Asia exhibit exceptional compatibility for *M. phaseolina*. The fungus is predominantly found in the Old World; nevertheless, the results suggest that its habitat is also suitable in temperate regions of North America, southeastern South America, and western Australia.

The future predictive modeling and calibration maps indicate the spread of the fungus towards Eastern Europe, specifically Russia and Ukraine. The minimum temperature of the coldest month (bio_6) facilitated the fungus’s adaptability to low temperatures (3.3°C to 10°C). This disruption may result from the adverse effects of global warming, which will certainly jeopardize the future production of numerous commodities, including *Triticum aestivum* L. (wheat) and *Zea mays* L. (maize) in Russia and Ukraine, representing over 30% of global wheat trade (US Department of Agriculture World Agricultural Supply and Demand Estimates (WASDE), 2023). Conversely, the fungus will forfeit the majority of its habitats in Africa due to the anticipated high temperature increase in equatorial regions and other areas that would be above the fungus’s tolerance threshold. In the New World, the prevalence of fungus remains largely unchanged. These maps and data will assist specialists in plant pathology and management in assessing the future distribution risk of *M. phaseolina*.

The current study provides updated and detailed maps about the global prevalence of *M. phaseolina* under changing climate through the present and future periods, where this work is considered the first modeling to anticipate the global prevalence of this fungus using the robust predictive powers of MaxEnt and DIVA-GIS. In comparison to other works related to the distribution of fungi, (Alkhalifah et al., 2023). reported differences in habitat suitability between the current and future distributions of *Fusarium oxysporum*, especially in Europe, due to global warming. The most effective climatological parameter of *F. oxysporum* distribution was the annual mean temperature (Bio-1), which disagreed with our work. Also, the annual mean temperature (bio-1) formed the most contributed bioclimatological parameter to *Aspergillus niger* distribution (Alkhalifah et al., 2022). On the other hand, *A. niger* will gain new habitat in several parts of the world (the Eastern part of Europe and Central Asia) where it will form emergence medical and agricultural issues (Alkhalifah et al., 2022). From the limited works about the distribution of fungi responding to climate change, Europe will be a good habitat for emerging fungi. Also, the study of (Wu et al., 2024) utilized the MaxEnt model and ArcGIS to map suitable habitats for the invasive weeds *Avena sterilis* and *Avena ludoviciana* in Asia, highlighting the significant risk they pose to dryland crops under climate change.

Considering other environmental covariates involving human population, land cover, host animal distribution, and vegetation index could aid in ameliorating them (Hosni et al., 2022). However, the decreasing of future data on these variables may limit their utility in researching the effect of climate change on present distribution models. Despite it all, our research contributes to a greater understanding of the current and future distribution of *M. phaseolina* worldwide. The models generated in this study analyzed the impact of climate change on the existing and future prevalence of the fungus using bioclimatological factors.

## 5 Conclusions

When it comes to forecasting the future distribution of dangerous fungus, SDM is an important and powerful tool. Based on the findings of this research, it was determined that *M. phaseolina* will continue to spread towards Eastern Europe and some regions of South America, whereas it will be eradicated from certain locations in Africa and Asia. The current findings of this research serve as a cautionary tale about the ways in which climate change can potentially alter the geographic distribution of *M. phaseolina* across the globe. Therefore, in order to improve our ability to predict the spread of this fungus, we need to conduct additional research on it, with a particular focus on the habitat suitability that influences its invasion pattern. In order to combat the phenomenon of fungal adaptability to a variety of environmental conditions, it is of the utmost importance to create advanced monitoring and control measures.

## Data Availability

The original contributions presented in the study are included in the article/**Supplementary Material**, further inquiries can be directed to the corresponding author/s.

## References

[B1] Aguilar-Paredes A. Valdés G. Araneda N. Valdebenito E. Hansen F. Nuti M. (2023). Microbial community in the composting process and its positive impact on the soil biota in sustainable agriculture. Agronomy 13, 542. doi: 10.3390/agronomy13020542

[B2] Akhtar K. P. Sarwar G. Arshad H. M. I. (2011). Temperature response, pathogenicity, seed infection and mutant evaluation against *Macrophomina phaseolina* causing charcoal rot disease of sesame. Arch. Phytopathol. Plant Prot. 44, 320–330. doi: 10.1080/03235400903052945

[B3] Alkhalifah D. H. M. Damra E. Khalaf S. M. H. Hozzein W. N. (2022). Biogeography of black mold Aspergillus Niger: Global situation and future perspective under several climate change scenarios using MaxEnt modeling. Diversity 14, 845. doi: 10.3390/d14100845

[B4] Alkhalifah D. H. M. Damra E. Melhem M. B. Hozzein W. N. (2023). Fungus under a Changing Climate: Modeling the Current and Future Global Distribution of *Fusarium oxysporum* Using Geographical Information System Data. Microorganisms 11, 468. doi: 10.3390/microorganisms11020468 36838433 PMC9967672

[B5] Allouche O. Tsoar A. Kadmon R. (2006). Assessing the accuracy of species distribution models: Prevalence, kappa and the true skill statistic (TSS). J. Appl. Ecol. 43, 1223–1232. doi: 10.1111/j.1365-2664.2006.01214.x

[B6] Ancin-Murguzur F. J. Barbero-López A. Kontunen-Soppela S. Haapala A. (2018). Automated image analysis tool to measure microbial growth on solid cultures. Comput. Electron. Agric. 151, 426–430. doi: 10.1016/j.compag.2018.06.031

[B7] Balakrishnan B. Nandakumar N. Sebastin S. Kareem K. A. A. (2018). “Species distribution models (SDM)—a strategic tool for predicting suitable habitats for conserving the target species: GIS and special distribution modelling (SDM),” in Association IRM, editor. Environmental Information Systems: Concepts, Methodologies, Tools, and Applications (IGI Global, Hershey, PA, USA), 555–568. doi: 10.4018/978-1-5225-7033-2.ch023

[B8] Bashir M. R. (2017). Impact of global climate change on charcoal rot of sesame caused by *macrophomina phaseolina* . J. Hortic. 4, e106. doi: 10.4172/2376-0354.1000e106

[B9] Borer E. T. Laine A.-L. Seabloom E. W. (2016). A multiscale approach to plant disease using the metacommunity concept. Annu. Rev. Phytopathol. 54, 397–418. doi: 10.1146/annurev-phyto-080615-095959 27296140

[B10] Bradie. J. Leung B. (2017). A quantitative synthesis of the importance of variables used in MaxEnt species distribution models. J. Biogeogr. 44, 1344–1361. doi: 10.1111/jbi.12894

[B11] Centers for Disease Control and Prevention (CDC). (2022). One Health Basics. Available online at: https://www.cdc.gov/onehealth/basics/ (Accessed February 5, 2023).

[B12] Cesondes I. Cseh A. Taller J. Poczai P. (2012). Genetic diversity and effect of temperature and pH on the growth of *Macrophomina phaseolina* isolates from sunflower fields in Hungary. Mol. Biol. Rep. 39, 3259–3269. doi: 10.1007/s11033-011-1094-6 21695429

[B13] Cesondes I. Kadlicsk S. Gaborjanyi R. Sandor K. Richard G. (2007). Growth of Macrophomina phaseolina isolates depend on different temperature Vol. 12 (Oradea City, Romania: Analele Universitaţii din Oradea, Fascicula: Protecţia Mediului), 31–34.

[B14] Chen K. Wang B. Chen C. Zhou G. (2022). MaxEnt modeling to predict the current and future distribution of *Pomatosace filicula* under climate change scenarios on the Qinghai–Tibet Plateau. Plants 11, 670. doi: 10.3390/plants11050670 35270140 PMC8912362

[B15] Das I. K. Fakrudin B. Arora D. K. (2008). RAPD cluster analysis and chlorate sensitivity of some Indian isolates of *Macrophomina phaseolina* from sorghum and their relationships with pathogenicity. Microbiol. Res. 163, 215–224. doi: 10.1016/j.micres.2006.05.006 16809028

[B16] Dell’Olmo E. Tripodi P. Zaccardelli M. Sigillo L. (2022). Occurrence of *Macrophomina phaseolina* on chickpea in Italy: Pathogen identification and characterization. Pathogens 11, 842. doi: 10.3390/pathogens11080842 36014963 PMC9415271

[B17] Deneu B. Joly A. Bonnet P. Servajean M. Munoz F. (2022). Very high-resolution species distribution modeling based on remote sensing imagery: How to capture fine-grained and large-scale vegetation ecology with convolutional neural networks? Front. Plant Sci. 13. doi: 10.3389/fpls.2022.839279 PMC9122285 35599901

[B18] Dietzel K. Valle D. Fierer N. U’Ren J. M. Barberán A. (2019). Geographical distribution of fungal plant pathogens in dust across the United States. Front. Ecol. Evol. 7. doi: 10.3389/fevo.2019.00304

[B19] Elith J. Kearney M. Phillips S. (2010). The art of modelling range-shifting species. Methods Ecol. Evol. 25, 330–342. doi: 10.1111/j.2041-210X.2010.00036.x

[B20] Fang X. Phillips D. Li H. Sivasithamparam K. Barbetti M. J. (2011). Comparisons of virulence of pathogens associated with crown and root diseases of strawberry in Western Australia with special reference to the effect of temperature. Sci. Hortic. 131, 39–48. doi: 10.1016/j.scienta.2011.09.025

[B21] Farr D. F. Rossman A. Y. Fungal Databases, U.S. National Fungus Collections, ARS, USDA. (2022). Available online at: https://nt.ars-grin.gov/fungaldatabases/ (Accessed October 21, 2022).

[B22] Fischer M. M. Nijkamp P. (1992). Geographic information systems and spatial analysis. Ann. Reg. Sci. 26, 3–17. doi: 10.1007/BF01581477

[B23] Food and Agriculture Organization of the United Nations (FAO) (2020). International year of plant health – protecting plants, Protecting life. Available online at: http://www.fao.org/plant-health-2020 (Accessed December 07, 2022).

[B24] Garcia S. N. Osburn B. I. Jay-Russell M. T. (2020). One health for food safety, food security, and sustainable food production. Front. Sustain. Food Syst. 4. doi: 10.3389/fsufs.2020.00001

[B25] GBIF.org (2022). GBIF Occurrence Download. doi: 10.15468/dl.f23yxn

[B26] Ghini R. Hamada E. Angelotti F. Costa L. B. Bettiol W. (2012). Research approaches, adaptation strategies, and knowledge gaps concerning the impacts of climate change on plant diseases. Trop. Plant Pathol. 37, 5–24. doi: 10.1590/S1982-56762012000100002

[B27] Ghosh T. Biswas M. K. Guin C. Roy P. (2018). A review on characterization, therapeutic approaches and pathogenesis of Macrophomina phaseolina. Plant Cell Biotechnol. Mol. Biol. 19, 72–84.

[B28] Goudarzi A. Banihashemi Z. Maftoun M. (2008). Effect of water potential on sclerotial germination and mycelial growth of *Macrophomina phaseolina. Phytopathol* . Mediterr. 47, 107–114. doi: 10.14601/Phytopathol_Mediterr-2613

[B29] Heringer G. Bueno M. L. Meira-Neto J. A. Matos F. A. Neri A. V. (2019). Can *Acacia mangium* and *Acacia auriculiformis* hinder restoration efforts in the Brazilian Atlantic Forest under current and future climate conditions? Biol. Invasions. 21, 2949–2962. doi: 10.1007/s10530-019-02024-7

[B30] Hosni E. M. Al-Khalaf A. A. Nasser M. G. Abou-Shaara H. F. Radwan M. H. (2022). Modeling the potential global distribution of honeybee pest, *Galleria mellonella* under changing climate. Insects 13, 484. doi: 10.3390/insects13050484 35621818 PMC9143048

[B31] Hosni E. M. Nasser M. G. Al-Ashaal S. A. Rady M. H. Kenawy M. A. (2020). Modeling current and future global distribution of *Chrysomya bezziana* under changing climate. Sci. Rep. 10, 4947. doi: 10.1038/s41598-020-61962-8 32188920 PMC7080715

[B32] Ibáñez A. Garrido-Chamorro S. Barreiro C. (2023). Microorganisms and climate change: A not so invisible effect. Microbiol. Res. 14, 918–947. doi: 10.3390/microbiolres14030064

[B33] Kaur S. Dhillon G. S. Brar S. K. Vallad G. E. Chand R. Chauhan V. B. (2012). Biology, economic importance and current diagnostic trends. Crit. Rev. Microbiol. 38, 136–151. doi: 10.3109/1040841X.2011.640977 22257260

[B34] Kaur S. Gonçalves-Vidigal M. C. Davidson J. Mysore K. S. Pandey A. K. (2023). Editorial: Disease and pest resistance in legume crops. Front. Plant Sci. 14. doi: 10.3389/fpls.2023.1166387 PMC10040850 36993859

[B35] Khambhati V. H. Abbas H. K. Sulyok M. Tomaso-Peterson M. Shier W. T. (2020). First report of the production of mycotoxins and other secondary metabolites by *Macrophomina phaseolina* (Tassi) Goid. isolates from soybeans (*Glycine max* L.) symptomatic with charcoal rot disease. J. Fungi 6, 332. doi: 10.3390/jof6040332 PMC7761776 33287215

[B36] Lodha S. Mawar R. (2020). Population Dynamics of Macrophomina phaseolina in Relation to disease management: A review. J. Phytopathol. 168, 1–17. doi: 10.1111/jph.12854

[B37] Lodha S. Sharma S. Aggarwal R. (2022). Inactivation of *Macrophomina phaseolina* propagules during composting and effect of composts on dry root rot severity and on seed yield of Clusterbean. Eur. J. Plant Pathol. 108, 253–261. doi: 10.1023/A:1015103315068

[B38] Makori D. M. Fombong A. T. Abdel-Rahman E. M. Nkoba K. Ongus J. Irungu J.. (2017). Predicting spatial distribution of key honeybee pests in Kenya using remotely sensed and bioclimatic variables: key honeybee pests distribution models. ISPRS Int. J. Geo-Inf. 6, 66. doi: 10.3390/ijgi6030066

[B39] Manici L. M. Caputo F. Cerato C. (1995). Temperature responses of isolates of *Macrophomina phaseolina* from different climatic regions of sunflower production in Italy. Plant Dis. 79, 834–838. doi: 10.1094/PD-79-0834

[B40] Marquez N. Giachero M. L. Declerck S. Ducasse D. A. (2021). *Macrophomina phaseolina*: General characteristics of pathogenicity and methods of control. Front. Plant Sci. 22. doi: 10.3389/fpls.2021.634397 PMC8100579 33968098

[B41] Merow C. Silander J. A. (2014). comparison of Maxlike and Maxent for modelling species distributions. Methods Ecol. Evol. 3, 215–225. doi: 10.1111/2041-210X.12152

[B42] Mihail J. D. Taylor S. J. (1995). Interpreting variability among isolates of *Macrophomina phaseolina* in pathogenicity, pycnidium production, and chlorate utilization. Canad. J. Bot. 73, 1596–1603. doi: 10.1139/b95-172

[B43] Mohammadi S. Ebrahimi E. Moghadam M. S. Bosso L. (2019). Modelling current and future potential distributions of two desert jerboas under climate change in Iran. Ecol. Inform. 52, 7–13. doi: 10.1016/j.ecoinf.2019.04.003

[B44] Mora C. McKenzie T. Gaw I. M. Dean J. M. von Hammerstein H. Knudson T. A.. (2022). Over half of known human pathogenic diseases can be aggravated by climate change. Nat. Clim. Change 12, 869–875. doi: 10.1038/s41558-022-01426-1 PMC9362357 35968032

[B45] Moritz M. A. Parisien M.-A. Batllori E. Krawchuk M. A. Van Dorn J. Ganz D. J.. (2012). Climate change and disruptions to global fire activity. Ecosphere 3, 1–22. doi: 10.1890/ES11-00345.1

[B46] Mulieri P. R. Patitucci L. D. (2019). Using ecological niche models to describe the geographical distribution of the myiasis-causing *Cochliomyia hominivorax* (Diptera: Calliphoridae) in southern South America. Parasitol. Res. 118, 1077–1086. doi: 10.1007/s00436-019-06267-0 30783861

[B47] Murad A. Khashoggi B. F. (2020). Using GIS for disease mapping and clustering in Jeddah, Saudi Arabia. ISPRS Int. J. Geo-Inf. 9, 328. doi: 10.3390/ijgi9050328

[B48] Mustafa H. K. Anwer S. S. Zrary T. J. (2023). Influence of pH, agitation speed, and temperature on growth of fungi isolated from Koya, Iraq. Kuwait J. Sci. 50 (4), 657–664. doi: 10.1016/j.kjs.2023.02.036

[B49] Nabil M. Zhang M. Bofana J. Wu B. Stein A. Dong T.. (2020). Assessing factors impacting the spatial discrepancy of remote sensing based cropland products: A case study in Africa. Int. J. Appl. Earth Obs. Geoinf. 85, 102010. doi: 10.1016/j.jag.2019.102010

[B50] Naeem M. Yuan X. Huang J. An J. (2018). Habitat suitability for the invasion of *Bombus terrestris* in East Asian countries: A case study of spatial overlap with local Chinese bumblebees. Sci. Rep. 8, 11035. doi: 10.1038/s41598-018-29414-6 30038323 PMC6056460

[B51] Nnadi N. E. Carter D. A. (2021). Climate change and the emergence of fungal pathogens. PloS Pathog. 17, 1–6. doi: 10.1371/journal.ppat.1009503 PMC8084208 33914854

[B52] Ortiz V. Chang H. X. Sang H. Jacobs J. Malvick D. K. Baird R.. (2023). Population genomic analysis reveals geographic structure and climatic diversification for *Macrophomina phaseolina* isolated from soybean and dry bean across the United States, Puerto Rico, and Colombia. Front. Genet. 14. doi: 10.3389/fgene.2023.1103969 PMC10282554 37351341

[B53] Pandey A. K. Basandrai A. K. (2020). Will *Macrophomina phaseolina* spread in legumes due to climate change? A critical review of current knowledge. J. Plant Dis. Prot. 128, 9–18. doi: 10.1007/s41348-020-00374-2

[B54] Pearson R. G. (2010). Species’ distribution modeling for conservation educators and practitioners. Lessons Conserv. 3, 54–89.

[B55] Phillips S. J. Anderson R. P. Schapire R. E. (2006). Maximum entropy modeling of species geographic distributions. Ecol. Modell. 190, 231–259. doi: 10.1016/j.ecolmodel.2005.03.026

[B56] Phillips S. J. Dudík M. (2008). Modeling of species distributions with Maxent: New extensions and a comprehensive evaluation. Ecography 31, 161–175. doi: 10.1111/j.0906-7590.2008.5203.x

[B57] Phillips S. J. Dudík M. Schapire R. E. (2022). Maxent Software for Modeling Species Niches and Distributions (Version 3.4.1). Available online at: http://biodiversityinformatics.amnh.org/open_source/maxent/ (Accessed December 5, 2022).

[B58] Remya K. Ramachandran A. Jayakumar S. (2015). Predicting the current and future suitable habitat distribution of Myristica dactyloides Gaertn. using MaxEnt model in the Eastern Ghats, India. Ecol. Eng. 9, 184–188. doi: 10.1016/j.ecoleng.2015.04.053

[B59] Rizzo D. M. Lichtveld M. Mazet J. A. K. Togami E. Miller S. A. (2021). Plant health and its effects on food safety and security in a One Health framework: four case studies. One Health Outlook 3, 6. doi: 10.1186/s42522-021-00038-7 33829143 PMC8011176

[B60] Runquist R. D. B. Lake T. Tiffin P. Moeller D. A. (2019). Species distribution models throughout the invasion history of Palmer amaranth predict regions at risk of future invasion and reveal challenges with modeling rapidly shifting geographic ranges. Sci. Rep. 9, 2426. doi: 10.1038/s41598-018-38054-9 30787301 PMC6382853

[B61] Saha A. Rahman S. Alam S. (2021). Modeling current and future potential distributions of desert locust *Schistocerca gregaria* (Forskål) under climate change scenarios using MaxEnt. J. Asia-Pac. Biodivers. 14, 399–409. doi: 10.1016/J.JAPB.2021.05.001

[B62] Samy A. M. Elaagip A. H. Kenawy M. A. Ayres C. F. Peterson A. T. Soliman D. E. (2016). Climate change influences on the global potential distribution of the mosquito Culex quinque fasciatus, vector of West Nile virus and lymphatic filariasis. PloS One 11, e0163863. doi: 10.1371/journal.pone.0163863 27695107 PMC5047650

[B63] Sarr M. P. Ndiaya M. Groenewald J. Z. Crous P. W. (2014). Genetic diversity in *Macrophomina phaseolina*, the causal agent of charcoal rot. Phytopathol. Mediterr. 53, 250–268. doi: 10.14601/Phytopathol_Mediterr-13736

[B64] Savary S. Bregaglio S. Willocquet L. Gustafson D. Mason D’Croz D. Sparks A.. (2017). Crop health and its global impacts on the components of food security. Food Secur. 9, 311–327. doi: 10.1007/s12571-017-0659-1

[B65] Savary S. Willocquet L. Pethybridge S. J. Esker P. McRoberts N. Nelson A. (2019). The global burden of pathogens and pests on major food crops. Nat. Ecol. Evol. 3, 430–439. doi: 10.1038/s41559-018-0793-y 30718852

[B66] Sax D. F. Early R. Bellemare J. (2013). Niche syndromes, species extinction risks, and management under climate change. Trends. Ecol. Evol. 28, 517–523. doi: 10.1016/j.tree.2013.05.010 23769712

[B67] Shabani F. Kumar L. Esmaeili A. (2014). Future Distributions of *Fusarium oxysporum* f. spp. in European, middle eastern and north African agricultural regions under climate change. Agric. Ecosyst. Environ. 197, 96–105. doi: 10.1016/j.agee.2014.08.005

[B68] Sohail M. Aqueel M. A. Ellis J. D. Raza A. M. Ullah S. (2020). Consumption, digestion, and utilization of beeswax by greater wax moths (*Galleria mellonella* L.*)* . J. Apic. Res. 59, 876–882. doi: 10.1080/00218839.2020.1765482

[B69] Taliei F. Safaie N. Aghajani M. A. (2013). Spatial distribution of *Macrophomina phaseolina* and soybean charcoal rot incidence using geographic information system (A case study in Northern Iran). J. Agr. Sci. Tech. 5, 1523–1536.

[B70] Tančić Živanov S. Dedić B. Dimitrijević A. Dušanić N. Jocić S. Miklič V.. (2019). Analysis of genetic diversity among *Macrophomina phaseolina* (Tassi) Goid. isolates from Euro-Asian countries. J. Plant Dis. Prot 126, 565–573. doi: 10.1007/s41348-019-00260-6

[B71] Tavanpour T. Sarafrazi A. Mehrnejad M. R. Imani S. (2019). Distribution modelling of *Acrosternum* spp. (*Hemiptera : Pentatomidae*) in south of Iran. Biologia 74, 1–9. doi: 10.2478/s11756-019-00266-3

[B72] US Department of Agriculture World Agricultural Supply and Demand Estimates (WASDE). (2023). Available online at: https://www.usda.gov/oce/commodity/wasde (Accessed February 2, 2023).

[B73] Veverka K. Palicová J. Křížková I. (2008). The incidence and spreading of Macrophomina phaseolina (Tassi) Goidanovich on sunflower in the Czech Republic. Plant Protect. Sci. 44, 127–137. doi: 10.17221/31/2008-PPS

[B74] Wang R. Li Q. He S. Liu Y. Wang M. Jiang G. (2018). Modeling and mapping the current and future distribution of *Pseudomonas syringae* pv. *actinidiae* under climate change in China. PloS One 13, e0192153. doi: 10.1371/journal.pone.0192153 29389964 PMC5794145

[B75] Warren D. L. Glor R. E. Turelli M. (2008). Environmental niche equivalency versus conservatism: quantitative approaches to niche evolution. Evolution 62, 2868–2883. doi: 10.1111/j.1558-5646.2008.00482.x 18752605

[B76] Wrather J. A. Anderson T. R. Arsyad D. M. Tan Y. Ploper L. D. Porta-Puglia A.. (2001). Soybean disease loss estimates for the top 10 soybean producing countries in 1998. Can. J. Plant Pathol. 23, 115–221. doi: 10.1080/07060660109506918 30870925

[B77] Wu B. M. van Bruggen A. H. C. Subbarao K. V. Pennings G. G. H. (2001). Spatial analysis of lettuce downy mildew using geostatistics and geographic information systems. Phytopath. 91, 134–142. doi: 10.1094/PHYTO.2001.91.2.134 18944386

[B78] Wu K. Wang Y. Liu Z. Huo W. Cao J. Zhao G.. (2024). Prediction of potential invasion of two weeds of the genus Avena in Asia under climate change based on Maxent. Sci. Total Environment. 950, 175192. doi: 10.1016/j.scitotenv.2024.175192 39111452

[B79] Yang X. Q. Kushwaha S. P. S. Saran S. Xu J. Roy P. S. (2013). Maxent modeling for predicting the potential distribution of medicinal plant, Justicia adhatoda L. @ in Lesser Himalayan foothills. Ecol. Eng. 51, 83–87. doi: 10.1016/j.ecoleng.2012.12.004

[B80] Yi Y. J. Cheng X. Yang Z. F. Zhang S. H. (2016). Maxent modeling for predicting the potential distribution of endangered medicinal plant (H. riparia Lour) Yunnan China. Ecol. Eng. 92, 260–269. doi: 10.1016/j.ecoleng.2016.04.010

[B81] Zurell D. Franklin J. König C. Bouchet P. J. Dormann C. F. Elith J.. (2020). A standard protocol for reporting species distribution models. Ecography. 43, 1261–1277. doi: 10.1111/ecog.04960

