# Supplementary material for: Impact of climate change on the potential global prevalence of Macrophomina phaseolina (Tassi) Goid. under several climatological scenarios
Source: Front Plant Sci. 2025 Apr 16;16:1512294. doi: 10.3389/fpls.2025.1512294 (PMC12040947; doi:10.3389/fpls.2025.1512294)
Supplement: Supplementary file 1 [file DataSheet1.zip › Table S1.pdf]

S1 Table. List of locations used for this study, with latitude, longitude, and sources.

Country	Region	Latitude	Longitude	Sources
Egypt	El-Behera, Housh Eisa	30.91361111	30.29444444	[1-3]
	El-Behera, Abo El-Matameer	30.91166667	30.17194444	
	El-Behera, Etay El-Barood	30.88583333	30.66583333	
	El-Behera, El-Dalangat	30.83083333	30.55194444	
	El-Behera, El-Mahmoudia	31.18361111	30.52444444	
	El-Behera, Kafr El-Dawar	31.13388889	30.13333333	
	Qalyubia	30.32916667	31.21666667	
	Beheira	30.84805556	30.34333333	
	Sharqia	30.5825	31.56944444	
	Dakahlia	31.16555556	31.49111111	
	Giza	30.01305556	31.20861111	
	Kafr El Sheikh	31.11055556	30.93861111	
	Faiyum	29.30833333	30.84277778	
	Beni Suef	29.06611111	31.09916667	
	Minya	28.08694444	30.76166667	
	Asyut	27.17805556	31.18583333	
	Sohag	26.55888889	31.74472222	
Iran	Joybar	36.63916667	52.90361111	[4,5]
	Qaemshar	36.46833333	52.86333333	
	Behshar	36.69861111	53.54222222	
	Kiakola	36.5825	52.81138889	
	Sari	36.56583333	53.05861111	
	Neka	36.65472222	53.30305556	
	Golestan Province	37.28972222	55.1375	
Senegal	Kaolack	13.80305556	-15.67777778	[6]
	Kaffrine	13.89777778	-15.3994444	
		13.99222222	-15.39	
	Saint-louis	16.11861111	-16.3644444	
		16.39722222	-15.8036111	
		16.53944444	-15.24	
	Diourbel	14.66111111	-16.5155556	
		14.82027778	-15.8244444	
	Tambacounda	13.83305556	-14.1630556	
		13.70361111	-13.9152778	
		13.52305556	-13.6075	
		13.67638889	-13.3161111	
	Louga	15.04861111	-15.6727778	
		15.37305556	-16.3447222	
		15.35388889	-16.2566667	

		15.77	-16.4047222	
		15.84888889	-16.1838889	
		15.57638889	-16.3497222	
	Thies	15.19555556	-16.8727778	
		15.07638889	-16.6441667	
		15.01638889	-16.2388889	
**Australia**	Lawes, queenland (Qld)	-27.5525	152.3352778	[7]
	Dimbulah, Qld	-17.1488889	145.1108333	
	Gatton, Qld	-27.5569444	152.2769444	
	Gumlu, Qld	-19.8788889	147.6861111	
	Mondure, Qld	-26.1819444	151.7752778	
	Kingaroy, Qld	-26.5397222	151.8430556	
	Walkamin, Qld	-17.1302778	145.4261111	
	Emerald, Qld	-23.5241667	148.1580556	
	Pemberton, Qld	-34.4455556	116.0330556	
	Kalbar, Qld	-27.9383333	152.6238889	
	Bioela, Qld	-24.4025	150.5122222	
	Clare, Qld	-33.8358333	138.6141667	
	Bowen, Qld	-20.0119444	148.2461111	
	Wamuran, Qld	-27.0394444	152.8638889	
	Lakeland, Qld	-15.8586111	144.8569444	
	Brookstead, Qld	-27.7586111	151.4480556	
	Jondaryn, Qld	-27.3675	151.5905556	
	Bundaberg, Qld	-24.8658333	152.3488889	
	Pampas, Qld	-27.7897222	151.4136111	
	Warra, Qld	-26.9302778	150.9202778	
	Kincora, Qld	-27.7886111	151.5261111	
	Inverell, NSW (new south wells)	-29.7738889	151.1113889	
	Grafton, NSW	-29.6813889	152.9380556	
	Warwick, NSW	-28.2136111	152.0305556	
	Piallamore, NSW	-31.1591667	151.0480556	
	Burroway, NSW	-33.0625	148.3236111	
	Edgeroi, NSW	-30.0827778	149.7938889	
	Tulloona, NSW	-28.9211111	150.0216667	
	Kununurra, WA (westeren australia)	-15.7783333	128.7438889	
	Carnarvon, WA	-24.8836111	113.6569444	
	Holt Rock, WA	-32.6775	119.4113889	
	Pingelly, WA	-32.5327778	117.0841667	
	Merredin, WA	-31.4830556	118.2830556	
	Kalannie, WA	-30.3666667	117.1208333	
	Wongan Hills, WA	-30.8936111	116.7175	

	Yealering, WA	-32.5936111	117.6244444	
	Katanning, WA	-33.6891667	117.555	
	Eneabba, WA	-29.8675	115.0227778	
	Geraldton, WA	-28.7761111	114.6111111	
	Cuballing, WA	-32.8183333	117.18	
	Circle Valley, WA	-33.0427778	121.8563889	
	Bayswater, WA	-31.9152778	115.9205556	
	Veľaty	48.52111111	21.66138889	
	Sv¨atuše	48.68333333	22.14277778	
	Lučenec	48.32861111	19.66888889	
	Fiľakovské Kováče	48.29194444	19.77611111	
	Rišňovce	48.36722222	17.89694444	
	Alekšince	48.36666667	17.94972222	
	Malanta	48.32805556	18.14611111	
	Malý Cetín	48.23666667	18.17611111	
	Dolný Štál	47.93611111	17.71638889	
	Dulov Dvor	47.78611111	18.16694444	
	Hul	48.10055556	18.26694444	
	Vinodol	48.19194444	18.21027778	
	Bíňa	47.91972222	18.63888889	
	Podhájska	48.10222222	18.33972222	
	Mojmírovce	48.20722222	18.06416667	
	Komárno	47.7625	18.12916667	
	Zbrojníky	48.11416667	18.70277778	
**Slovakia**	Kolíňany	48.35972222	18.19583333	**[8]**
	Neverice	48.36722222	18.27527778	
	Zlaté Moravce	48.385	18.39111111	
	Vráble	48.24333333	18.30861111	
	Cabaj-Čápor č. Pereš	48.21666667	17.99861111	
	Žihárec	48.07277778	17.88138889	
	Kamenín	47.88777778	18.645	
	Bruty	47.92333333	18.58027778	
	Borský Mikuláš	48.62888889	17.21222222	
	Bučany	48.41861111	17.70027778	
	Trnava	48.39416667	17.72138889	
	Pečeňady	48.48305556	17.71972222	
	Madunice	48.47666667	17.77972222	
	Trávnik	47.77611111	17.79833333	
	Sap	47.82166667	17.61472222	
	Lehnice	47.05138889	17.455	
	Bojničky	48.39083333	17.79805556	
	Šulekovo	48.42666667	17.77222222	
**Serbia**	Kuštin, Vojvodina province	45.19972222	20.13333333	

	Zrenjanin, Vojvodina province	45.38333333	20.39055556	[9]
	Bezdan, Vojvodina province	45.84722222	18.92388889	
	Deliblato, Vojvodina province	45.83861111	21.04111111	
	Rimski Šančevi, Vojvodina province	45.31111111	19.82472222	
	Kula, Vojvodina province	45.65611111	19.41805556	
	Pančevo, Vojvodina province	44.87055556	20.64777778	
	BačkaTopola, Vojvodina province	45.81277778	19.63	
	Bajmok, Vojvodina province	45.96666667	19.42388889	
	Crvenka, Vojvodina province	45.65833333	19.45583333	
	Novi Sad, Vojvodina province	45.23944444	19.8225	
**Brazil**	Realeza	-25.7680556	-53.53111111	[10]
	Londrina	-23.3197222	-51.1661111	
	Dourados	-22.2233333	-54.8122222	
	Fortaleza	-3.7325	-38.5269444	
	Uberaba	-19.7458333	-47.9338889	
	Ribeirão Preto	-21.1691667	-47.8108333	
	Santo Ant. Goiás	-16.4863889	-49.3108333	
	Barreiras	-12.1475	-44.9952778	
	Cristalina	-16.7638889	-47.6083333	
	Montividiu	-17.4466667	-51.1761111	
	Alvorada do Sul	-22.7802778	-51.2322222	
	Bela Vista Paraíso	-22.9955556	-51.1922222	
**Ghana**	Arigu	10.56194444	-0.848611111	[11]
	Asumsapeliga	10.96722222	-0.35694444	
	Binduri	10.97305556	-0.31583333	
	Feo	10.955	-0.79	
	Manga	11.015	-0.2625	
	Nafkluga	10.86888889	-0.38083333	
	Sakpari	10.92638889	-0.36861111	
	Sapeliga	11.07166667	-0.40694444	
	Chinchan	10.8675	-1.91805556	
	Lawra	10.65305556	-2.85694444	
	Silbelle	10.90611111	-2.00583333	
	Botanga	9.589444444	-1.0275	
	Damongo	9.045	-1.76805556	
	Nyankpala	9.392222222	-1.00305556	
	Kpasenkpe	10.44444444	-1.06916667	
	Yagaba	10.24805556	-1.28055556	
	Yendi	9.499722222	-0.02638889	
**Somalia**	Baidoa	3.113888889	43.65166667	[12]
	Burhakaba	2.798888889	44.07916667	

	Dinsoore	2.407222222	42.97083333	
	Quansadhere	2.815277778	42.75472222	
**Pakistan**	Mianwali	32.58388889	71.53694444	**[13]**
	Bhakkar	31.86194444	71.38222222	
	Layyah	30.96916667	70.94277778	
	Faisalabad	31.45027778	73.13472222	
	Muzaffargarh	30.07333333	71.18027778	
	Narowal	32.27277778	75.06111111	
	Sialkot	32.49444444	74.52277778	
	Chakwal	32.93277778	72.86277778	
	Islamabad	33.68416667	73.04777778	
	Rawalpindi	33.565	73.01666667	
	Kohat	33.58861111	71.44277778	
	Bannu	32.99083333	70.64527778	
	Dera Ghazi Khan	30.04888889	70.64527778	
	Dera Ismail Khan	31.8625	70.90166667	
**Ukraine**	Nova Kakhovka, Khersons' province	46.75138889	33.36777778	**[9]**
	Kherson, Khersons' province	46.63527778	32.61666667	
	Zaporizhia, Zaporizhia province	47.83861111	35.13944444	
	Kakhovka, Khersons' province	46.81166667	33.49	
**Bulgaria**	Dobrudzha, South region	43.54972222	28.13611111	**[9]**
	Burgas, North region	42.77611111	27.53138889	
**Romania**	Medgidia, East region RO2	44.25833333	28.4225	**[9]**
	Lunca, West region RO4	44.73527778	28.74583333	
**India**	Hyderabad	17.385	78.48666667	**[14-16]**
	Bhubaneswar	20.29583333	85.82444444	
	Ludhiana	30.90083333	75.85722222	
	Palampur	32.11083333	76.53611111	
	Mongrakalan	17.20027778	78.55027778	
	Jodhpur	26.23888889	73.02416667	
	Bijwaria	30.37694444	77.30305556	
	Rampur	28.79805556	79.02194444	
	Masa	29.26027778	78.14583333	
	Amravati	20.93194444	77.75222222	
	Kerala	10.85027778	76.27083333	
	Varanasi	25.3175	82.97388889	
	Cuddalore	11.74777778	79.77111111	
	Thiruvannamalai	12.22527778	79.07444444	
	Vellore	12.91638889	79.13222222	
**Venezuela**	Colonia Turen	9.211388889	-68.9463889	**[17]**
	El playon	9.128611111	-69.0288889	

	Villa Bruzual	9.302777778	-69.1202778	
	Chorrerones	9.127222222	-68.8886111	
	El playon	9.109444444	-69.0419444	
	Chorrerones	9.151111111	-68.9016667	
**Nigeria**	Nigeria	9.081944444	8.675277778	[15,18]
	Ibadan	7.3775	3.946944444	
	Mokwa	9.292777778	5.054444444	
	Onne	4.723611111	7.151388889	
	Kachia	9.873333333	7.955	
	Alabata	7.228333333	3.452777778	
	Kano	12.00194444	8.591944444	
	Abuja	9.076388889	7.398333333	
**Korea**	Wanju	35.89083333	127.2538889	[19,20]
	Hwaseong	37.19944444	126.8311111	
	Yeoncheon	38.09638889	127.0747222	
**China**	Jianshui, Yunnan Province	23.38	102.49	[21-24]
	Yulin, Shanxi Province	38.285	109.7344444	
	Fangshan County of Beijing	39.73444444	116.1916667	
	ChengMai	19.73833333	110.0047222	
	Jianshui	23.38	102.49	
	Shanghai	31.23027778	121.4736111	
**Argentina**	Tucuman, Puesto del Medio	-26.78972222	-64.6658333	[25,26]
	Tucuman, La Cocha	-27.7677778	-65.5838889	
	Tucuman, La Cruz	-29.1780556	-56.6377778	
	Tucuman, La Virginia	-27.3758333	-55.8986111	
	Tucuman, San Agustín	-31.9775	-64.375	
	Santiago del Estero, Rapelli	-26.5583333	-64.5052778	
	Santiago del Estero, Arenales	-34.2747222	-61.28	
	Salta, General Mosconi	-22.5958333	-63.8113889	
	Salta, Metán	-25.4941667	-64.9719444	
	Salta, Las Lajitas	-24.7272222	-64.1955556	
	Salta, Pichanal, Orán	-23.1283333	-64.3216667	
	Buenos Aires Province	-37.20166667	-59.84083333	
**USA**	Tennessee	35.64305556	-88.8041667	[15,27]
	Illinois	39.58472222	-88.1527778	
	Mississippi	31.66972222	-91.0313889	
		32.27527778	-90.5222222	
		32.78	-88.7425	
		34.7525	-89.9136111	
		34.79916667	-89.5427778	
		34.56555556	-88.5588889	
		34.3825	-89.4583333	
		33.74416667	-88.4977778	

		33.78666667	-88.8447222	
		34.04472222	-90.7372222	
		33.595	-90.8261111	
		33.57583333	-90.2458333	
	Indiana	40.26694444	-86.1347222	
	Kentucky	37.83916667	-84.27	
	Georgia	32.16555556	-82.9	
	Florida	27.66472222	-81.5155556	
	Iowa	41.87777778	-93.0975	
	Minnesota, Zumbrota	44.29388889	-92.6688889	
	California	36.77805556	-119.417778	
**Czech Republic**	Žatec	50.32694444	13.54555556	[28]
	west and east of prague	50.08583333	14.51805556	
	South Moravia, Lednice	48.79972222	16.80333333	
**Turkey**	Ordu province, Altinordu	40.9675	37.88972222	[9,29]
	Ordu province, Fasta	41.00694444	37.52805556	
	Ordu province, Persemble	41.04111111	37.76388889	
	Edirne, Thrace region	41.67833333	26.595	
**Japan**	Nagano	36.64833333	138.195	[15]
	Kanagawa	35.49111111	139.2838889	
**Benin**	Benin	9.3075	2.315833333	[15]
**France**	Aquitaine	44.7	-0.299444444	[15]
**Israel**	Israel	31.04583333	34.85138889	[15]
**Greece**	Amyndeon	40.69027778	21.67972222	[15]
**Malaysia**	Penang	5.413888889	100.3286111	[30]
**Libya**	Tripoli	32.88694444	13.19111111	[31]
**Spain**	Huelva	37.25	-6.95	[9,32]
	La Lusiana, Andalusia	37.49027778	-5.3	
**Chile**	San Pedro-Melipilla	-33.89444444	-71.45583333	[32,33]
	Curacavi Valley	-33.38833333	-71.0225	
**Myanmar (Burma)**	Yezin	19.83638889	96.27194444	[14]
**Tunisia**	Cap Bon peninsula	36.75	10.75	[34]
**Mexico**	Sonora, Hermosillo	29.02805556	-111.0561111	[35]

**Italy**	Salerno province, Campania region	40.42861111	15.21944444	[36]
**Bangladesh**	Sylhet	24.9	91.9	[37]
**Paraguay**	Guayaibi	-24.5	-56.6	[37]
	Kressburgo	-26.3	-55.1	
	Calle	-24.5	-56.6	
	Laurel	-24.6	-54.9	
	Misiones	-26.9	-55.9	
**Canada**	British columbia	51.7	-128.1	[37]
**Germany**	Essen	51.3	7.2	[37]
**Colombia**	Palmira	3.5	-76.3	[37]
	Colinas	11.3	-74	
**Tanzania**	Kitu	-6.9	37.4	[37]
**Cyprus**	Peyai	34.8	32.4	[37]
**Zimbabwe**	Matetsi	-18.1	25.5	[37]
**Oman**	Ibra	22.9	58.9	[37]
**Côte d'Ivoire**	Ono	5.3	-3.5	[37]
	Merimeri	9.3	-6.4	
**Hungary**	Kerekegyhaza	46.9	19.4	[37]
	Heviz	46.8	17.2	
	Rem	46.2	19	
**Antarctica**	Antarctica	-66.4	110.7	[37]
**New Zealand**	Gisborne	-38.7	178	[37]
	Puketapu	-39.5	176.8	
	Palmerston N	-40.4	175.6	
	Motueka	-41.1	173	
	Brightwater	-41.4	173.1	
	Victoria	-36.8	147.8	
	Levin	-40.6	175.3	
**South Africa**	Hogback	-32.6	26.9	[37]
	Boshoek	-25.5	27.1	
	Malvern	-26.2	28.1	
	Simonsberg	-33.9	18.9	
	Vaalkrans	-23.7	30.1	
	Glen Avon	-29.1	30.6	

	Bellville	-33.9	18.6	
	Roodeplaat	-25.6	28.4	
	Buffelshoek	-25.7	27.2	
**Switzerland**	Breil	46.8	9.1	[37]
**Philippines**	Calindacan	9.3	123.3	[37]
**Uganda**	Kakinzi	0.9	32.3	[37]
**Congo**	Kwilu Ngongo	-5.5	14.9	[37]
**Madagascar**	Toamasina	-18.1	49.4	[37]

References

1. Omar, M.R.; Abd-Elsalam, K.A.; Aly, A.A. et al. Diversity of *Macrophomina phaseolina* from cotton in Egypt: Analysis of pathogenicity, chlorate phenotypes, and molecular characterization. *J Plant Di.s Prot.* **2007**, *114*, 196–204. https://doi.org/10.1007/BF03356219

2. Aboshosha, S.S.; Attaalla, S.I.; El-Korany, A.E.; El-Argawy, E. Characterization of *Macrophomina phaseolina* isolates affecting sunflower growth in El-Behera governorate, Egypt. *Int. J. Agric. Biol.* **2007**, *9*, 807–815.

3. Yousef, H. Pathogenic Variation and Molecular Characterization of *Macrophomina phaseolina*, the Cause of Sesame Charcoal Rot. *Egypt. J. Phytopathol.* **2021**, *49*, 151-165. DOI: 10.21608/ejp.2021.77853.1035

4. Taliei, F.; Safaie, N.; Aghajani, M.A. Spatial Distribution of *Macrophomina phaseolina* and Soybean Charcoal Rot Incidence Using Geographic Information System (A Case Study in Northern Iran). *J. Agr. Sci. Tech.* **2013**, *15*, 1523-1536.

5. Rayatpanah, S.; Nanagulyan, G.; Seyed, V.; Razavi, M. Pathogenic and Genetic Diversity among Iranian Isolates of *Macrophomina phaseolina*. *Chil. J. Agric. Res.* **2012**, *72*, 40-44. https://dx.doi.org/10.4067/S0718-58392012000100007.

6. Starr, M.; Ndiaye, M.; Groenewald, L.; Crous, P. Genetic diversity in *Macrophomina phaseolina*, the causal agent of charcoal rot. *Phytopathol. Mediterr.* **2014**, *53*, 250-268. DOI: 10.14601/Phytopathol_Mediterr-13736.

7. Poudel, B.; Shivas, R.G.; Adorada, D.L. et al. Hidden diversity of *Macrophomina* associated with broadacre and horticultural crops in Australia. *Eur. J. Plant. Pathol.* **2021**, *161*, 1–23. https://doi.org/10.1007/s10658-021-02300-0

8. Bokor, P. *Macrophomina phaseolina* causing a charcoal rot of sunflower through Slovakia. *Biologia* **2007**, *62*, 136–138. DOI: 10.2478/s11756-007-0020-9

9. Tančić Živanov, S., Dedić, B.; Dimitrijević, A. et al. Analysis of genetic diversity among *Macrophomina phaseolina* (Tassi) Goid. isolates from Euro-Asian countries. *J. Plant. Dis. Prot.* **2019**, *126*, 565–573. https://doi.org/10.1007/s41348-019-00260-6

10. Almeida, A.M.R.; Abdelnoor, R.V.; Arias, C.A.A.; Carvalho, V.P; Jacoud, D.S.; Marin, S.R.R.; Benato, L.C.; Pinto, M.C.; Caravalho, C.G.P. Genotypic diversity among brazilian isolates of *Macrophomina phaseolina* revealed by RAPD. *Fitopatol. Bras.* **2003**, *28*, 279-285. https://doi.org/10.1590/S0100-41582003000300009.

11. Lamini, S.; Cornelius, E.W.; Kusi, F.; Danquah, A.; Attamah, P.; Mukhtaru, Z.; Awuku, J.F.; Mensah, G. Prevalence, incidence and severity of a new root rot disease of cowpea caused by *Macrophomina phaseolina* (Tassi) Goid in Northern Ghana. *West Afr. J. Appl. Ecol.* **2020**, *28*, 140 – 154.

12. Gray, F.A.; Mihail, J.D.; Lavigne, R.J. et al. Incidence of charcoal rot of sorghum and soil populations of *Macrophomina phaseolina* associated with sorghum and native vegetation in Somalia. *Mycopathologia* **1991**, *114*, 145–151. https://doi.org/10.1007/BF00437203

13. Iqbal, U.; Mukhtar, T. Morphological and pathogenic variability among *Macrophomina phaseolina* isolates associated with mungbean (*Vigna radiata* L.) Wilczek from Pakistan. *Sci. World J.* **2014**, *15*, 2014:950175. doi: 10.1155/2014/950175.

14. Pandey, A.K.; Yee, M.; Win, M.M.; Lwin, H.M.M.; Adapala, G.; Rathore, A.; Sheu Z.M.; Nair, R.M. Identification of new sources of resistance to dry root rot caused by *Macrophomina phaseolina* isolates from India and Myanmar in a mung bean mini-core collection. *Crop Prot.* **2021**, *143*, 105569. https://doi.org/10.1016/j.cropro.2021.105569

15. Kaur**,** S.; Dhillon, G.S.; Brar, S.K.; Vallad, G.E.; Chand, R.; Chauhan, V.B. Emerging phytopathogen *Macrophomina phaseolina*: biology, economic importance and current diagnostic trends. *Crit. Rev. Microbial.* **2012**, *38*, 136-51. doi: 10.3109/1040841X.2011.640977

16. Mohanapriya, R.; Naveenkumar, R.; Balabaskar, P. Survey, Virulence and Pathogenicity of Root Rot Incidence of Cowpea in Selected Districts of Tamilnadu caused by *Macrophomina phaseolina* (Tassi.) Goid. *Int. J. Curr. Microbiol. App. Sci.* **2017**, *6*, 694-705. doi: https://doi.org/10.20546/ijcmas.2017.603.080

17. Martínez-Hilders, A.; Laurentin, H. Phenotypic and molecular characterization of *Macrophomina phaseolina* (Tassi) Goid. coming from the sesame production zone in Venezuela. *Bioagro* **2012**, *24*, 187-196.

18. Amusa N.A.; Okechukwu R.U.; Akinfenwa B. Reactions of cowpea to infection by *Macrophomina phaseolina* isolates from leguminous plants in Nigeria. *Afr. J. Agric. Res.* **2007**, *2*, 073-075.

19. Kim, S.G.; Kim, T.B.; Lee, O.J. First Report of *Macrophomina phaseolina* Causing Charcoal Rot in Bottle Gourd in Korea. *Kor. J. Mycol.* **2021**, *49*, 399-403. https://doi.org/10.4489/KJM.20210037

20. Kim, H.T.; Ko, Y.M; Choi, J.; Lee, Y.H. First Report of Charcoal Rot Caused by *Macrophomina phaseolina* on *Glycine max* in Korea. *Res. Plant Dis.* **2020**, *26*, 29-37. https://doi.org/10.5423/RPD.2020.26.1.29

21. Sun, S.L.; Zhu, Z.D.; Duan, C.X.; Zhao, P.; et al. First Report of Charcoal Rot Caused by *Macrophomina phaseolina* on *Faba* Bean in China. *Plant Dis.* **2019**, *103*, 1415.

22. Sun, S.; Wang, X.; Zhu, Z.; Wang, B.; Wang, M. Occurrence of Charcoal Rot Caused by *Macrophomina phaseolina*, an Emerging Disease of Adzuki Bean in China. *J. Phytopathol.* **2016**, *164*, 212–216. doi: 10.1111/jph.12413.

23. Sun, X.D.; Cai, X.L.; Pang, Q.Q; Zhou, M.; Zhang, W.; Chen, Y.S.; Bian, Q. "First Report of *Macrophomina phaseolina* Causing Root Rot of Curcuma longa in China" *Plant Dis.* **2020**, *104*, 3261. doi: 10.1094/PDIS-12-19-2734-PDN

24. Wu, H.; Li, C.; Chakraborti, P.; Guo, Z.; Peng. B.; Gu, W.; Kang, B.; Gu, Q. First Report of Watermelon Charcoal Rot (*Macrophomina phaseolina*) in China. *Plant Dis.* **2022,** *106*,1521. doi: 10.1094/PDIS-07-21-1362-PDN.

25. Gaetán, S.A.; Fernandez, L.; Madia, M. Occurrence of Charcoal Rot Caused by *Macrophomina phaseolina* on Canola in Argentina. *Plant Dis.* **2006**, *90*, 524. doi: 10.1094/PD-90-0524A.

26. Sebastian, R.; Gabriel, R.V.; Alemu, A.; Renee, S.A.; Victoria, G.; Vicente, D.L et al. Disease incidence of charcoal rot (*Macrophomina phaseolina*) on soybean in north-western Argentina and genetic characteristics of the pathogen. *Can. J. Plant Pathol.* **2018**, *40*, 423-433, DOI: 10.1080/07060661.2018.1484390

27. Khambhati, V.H.; Abbas, H.K.; Sulyok, M.; Tomaso-Peterson, M.; Shier, W.T. First Report of the Production of Mycotoxins and Other Secondary Metabolites by *Macrophomina phaseolina* (Tassi) Goid. Isolates from Soybeans (*Glycine max* L.) Symptomatic with Charcoal Rot Disease. *J. Fungi* **2020**, *6*, 332. doi: 10.3390/jof6040332.

28. Veverka, K.; Palicová, J.; Křížkovám I. The incidence and spreading of *Macrophomina phaseolina* (Tassi) Goidanovich on sunflower in the Czech Republic. *Plant Protect. Sci.* **2008**, *44*, 127–137.

29. Türkkan, M.; Benli, H.İ.; Yılmaz, Ö. et al. First report of charcoal rot caused by *Macrophomina phaseolina* on kiwifruit in Turkey. *J. Plant. Pathol.* **2020**, *102*, 535. https://doi.org/10.1007/s42161-019-00428-x.

30. Huda-Shakirah, A.R.; Kee, Y.J.; Hafifi, A.B.M.; Mohamad, N.N.; Zakaria, L., M.H. Identification and Characterization of *Macrophomina phaseolina* Causing Leaf Blight on White Spider Lilies (*Crinum asiaticum* and *Hymenocallis littoralis*) in Malaysia. *Mycobiology*. **2019,** *47*, 408-414. doi: 10.1080/12298093.2019.1682448.

31. Abied, M.E.; Duzan, H.M. First Report of *Macrophomina phaseolina* Causing Crown and Root Rot of Strawberry in Tripoli, Libya. *J. Appl. Plant Prot.* **2020**, *9*, 83-84.

32. Sánchez, S.; Chamorro, M.; Henríquez, J.L. et al. Genetic and biological characterization of *Macrophomina phaseolina* (Tassi) Goid. causing crown and root rot of strawberry. *Chil. J. Agric. Res.* **2017**, *77*, 325–331. https://doi.org/10.4067/S0718-58392017000400325.

33. Jacob, C.J.; Krarup, C.; Díaz, G.A.; Latorre, B.A. A severe outbreak of charcoal rot in cantaloupe melon caused by *Macrophomina phaseolina* in Chile. *Plant Dis.* **2013**, *97*, 141-142. DOI:10.1094/PDIS-06-12-0588-PDN.

34. Hajlaoui, M.R.; Mnari-Hattab, M.; Sayeh, M.; Zarrouk, I.; Jemmali, A.; Koike, S.T. First report of Macrophomina phaseolina causing charcoal rot of strawberry in Tunisia. *New Dis. Rep.* **2015**, 14. DOI:10.5197/j.2044-0588.2015.032.014

35. Martínez-Salgado, S.J.; Romero-Arenas, O.; Morales-Mora, L.A.; Luna-Cruz, A.; Rivera-Tapia, J.A.; Silva-Rojas, H.V.; Andrade-Hoyos P. First Report of *Macrophomina phaseolina* Causing Charcoal Rot of Peanut (*Arachis hypogaea* L.) in Mexico. *Plant Dis.* **2021,** Mar 23. doi: 10.1094/PDIS-02-21-0337-PDN.

36. Dell'Olmo, E.; Tripodi, P.; Zaccardelli, M.; Sigillo, L. Occurrence of *Macrophomina phaseolina* on Chickpea in Italy: Pathogen Identification and Characterization. *Pathogens* **2022**, *11*, 842. https://doi.org/10.3390/pathogens11080842.

37. GBIF.org (28 October 2022) GBIF Occurrence Download https://doi.org/10.15468/dl.f23yxn
