# Supplementary material for: Impact of climate change on the potential global prevalence of Macrophomina phaseolina (Tassi) Goid. under several climatological scenarios
Source: Front Plant Sci. 2025 Apr 16;16:1512294. doi: 10.3389/fpls.2025.1512294 (PMC12040947; doi:10.3389/fpls.2025.1512294)
Supplement: Supplementary file 1 [file DataSheet1.zip › Table S2.pdf]

Table S2. Climatic variables used for predicting the biogeographic distribution of *M. phaseolina*.

Variable Code	Variable Description	Unit
Bio_1	Annual mean air temperature	^{0}C
Bio_2	Mean diurnal temperature range	^{0}C
Bio_3	Isothermality	^{0}C
Bio_4	Temperature seasonality	^{0}C
Bio_5	Max temperature of warmest period	^{0}C
Bio_6	Min temperature of coldest period	^{0}C
Bio_7	Temperature annual range	^{0}C
Bio_8	Mean temperature of wettest quarter	^{0}C
Bio_9	Mean temperature of driest quarter	^{0}C
Bio_10	Mean temperature of warmest quarter	^{0}C
Bio_11	Mean temperature of coldest quarter	^{0}C
Bio_12	Annual precipitation	mm
Bio_13	Precipitation of wettest period	mm
Bio_14	Precipitation of driest period	mm
Bio_15	Precipitation seasonality	mm
Bio_16	Precipitation of wettest quarter	mm
Bio_17	Precipitation of driest quarter	mm
Bio_18	Precipitation of warmest quarter	mm
Bio_19	Precipitation of coldest quarter	mm
