# Supplementary figures and images for: Impact of climate change on the potential global prevalence of Macrophomina phaseolina (Tassi) Goid. under several climatological scenarios
Source: Front Plant Sci. 2025 Apr 16;16:1512294. doi: 10.3389/fpls.2025.1512294 (PMC12040947; doi:10.3389/fpls.2025.1512294)

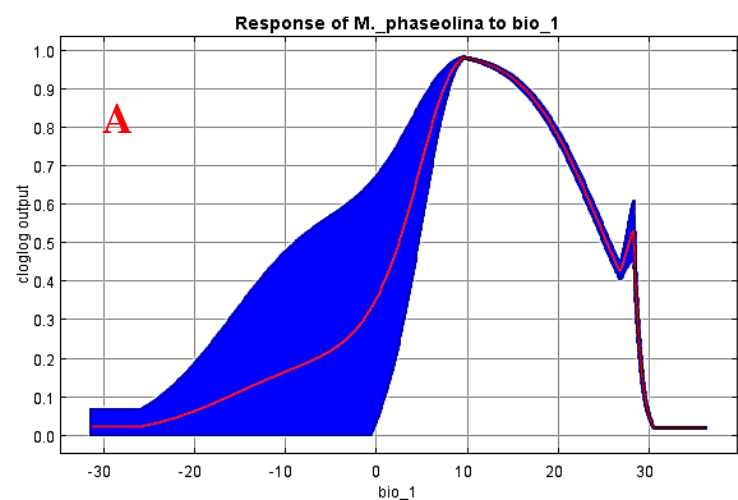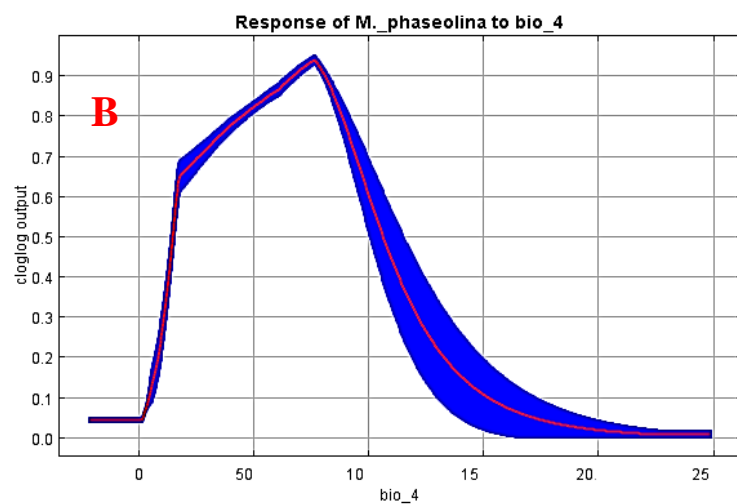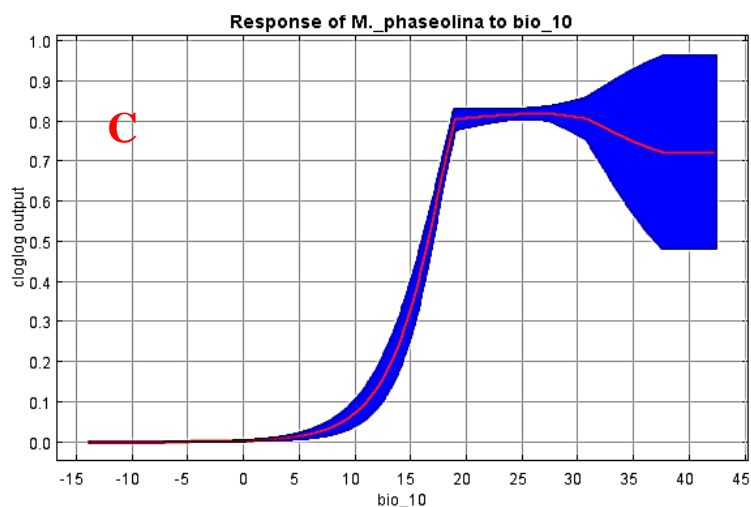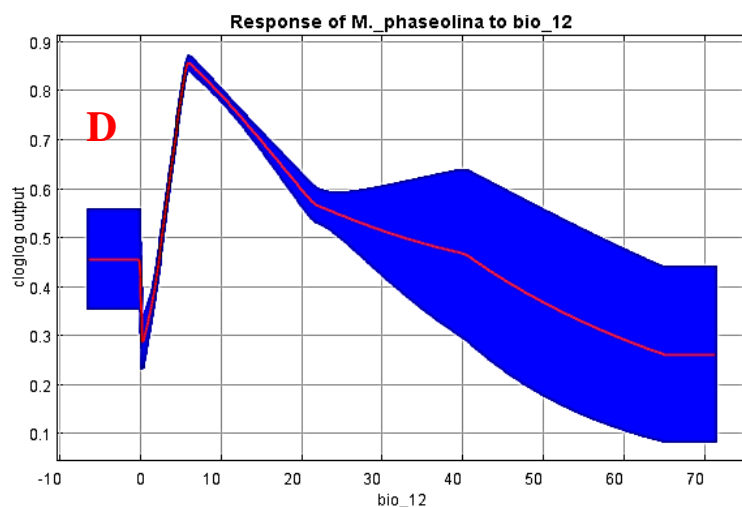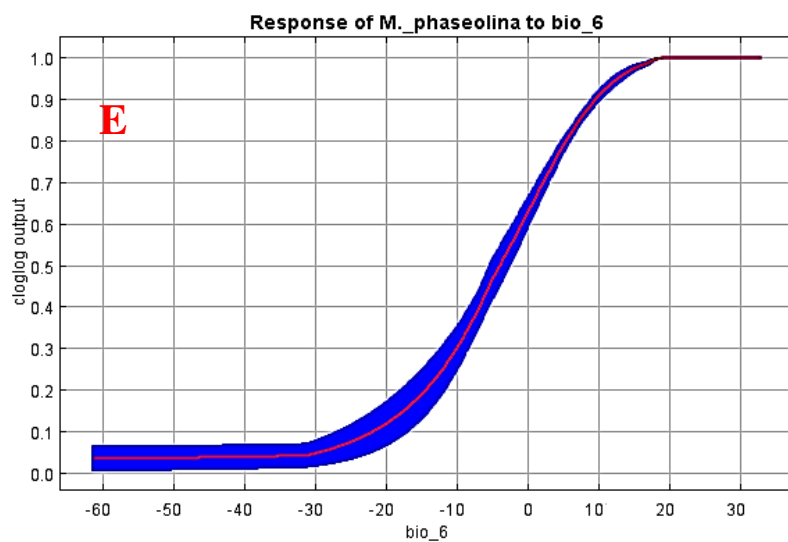

**S1 Figure.** *Macrophomina phaseolina* response curves in relation to: A, bio\_1; B, bio\_4; C, bio\_10; D, bio\_12; and E, bio\_6.

Supplement: Supplementary file 1 [file DataSheet1.zip › Figure S1.pdf]

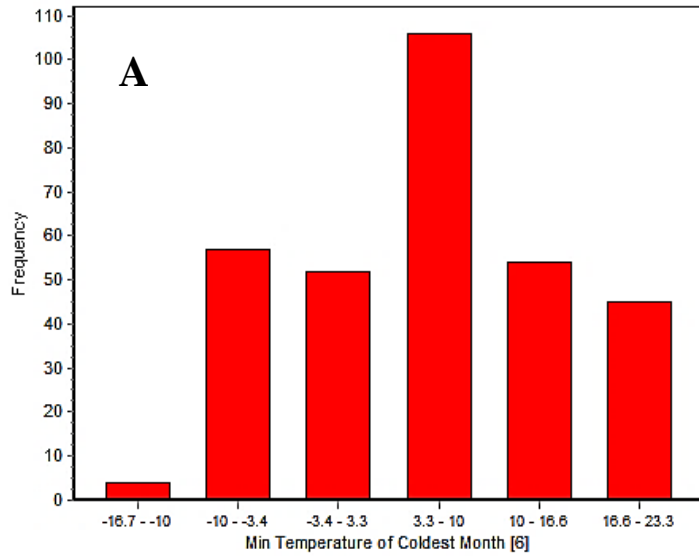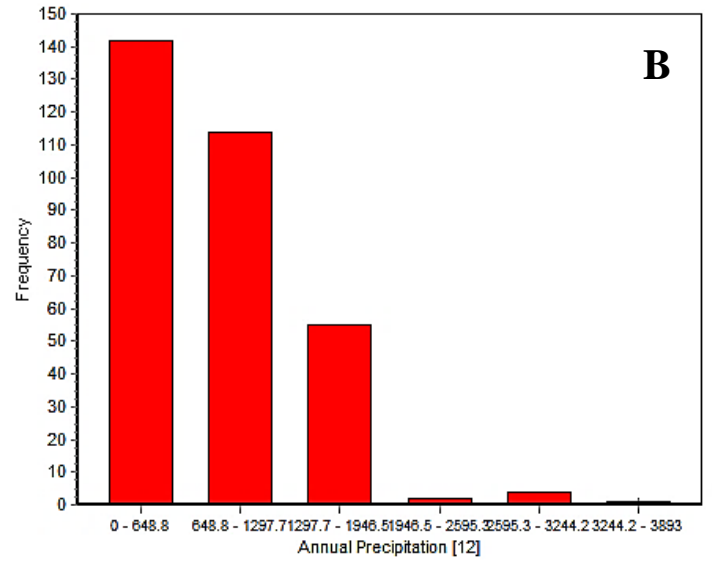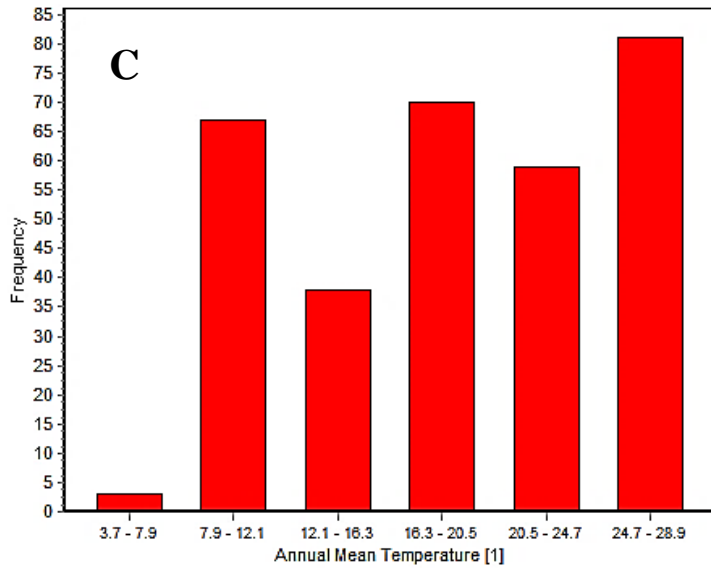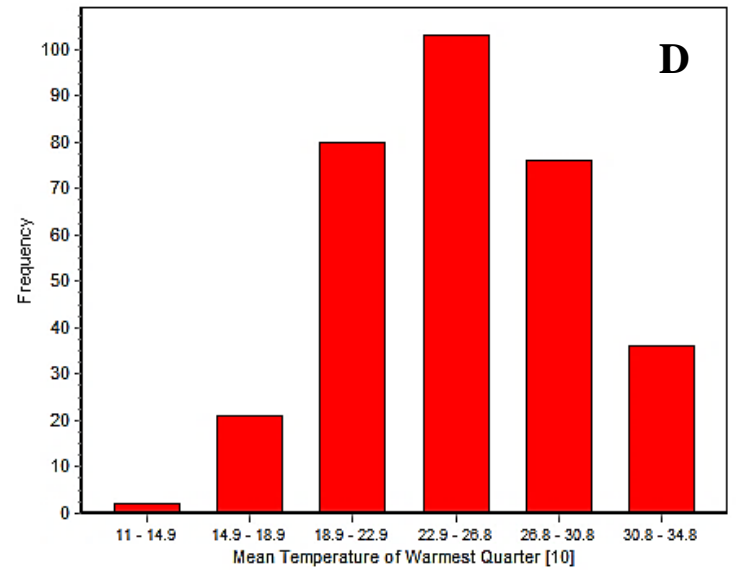

**S2 Figure.** *Macrophomina phaseolina* frequency analysis in relation to: A, bio\_6; B, bio\_12; C, bio\_1; and D, bio\_10.

Supplement: Supplementary file 1 [file DataSheet1.zip › Figure S2.pdf]
